# Pseudo five-component synthesis of 2,5-di(hetero)arylthiophenes via a one-pot Sonogashira–Glaser cyclization sequence

**DOI:** 10.3762/bjoc.7.174

**Published:** 2011-11-04

**Authors:** Dominik Urselmann, Dragutin Antovic, Thomas J J Müller

**Affiliations:** 1 Institut für Organische Chemie und Makromolekulare Chemie, Heinrich-Heine-Universität Düsseldorf, Universitätsstr. 1, D-40225 Düsseldorf, Germany

**Keywords:** C–C coupling, copper, multicomponent reactions, palladium, thiophenes

## Abstract

Based upon a consecutive one-pot Sonogashira–Glaser coupling–cyclization sequence a variety of 2,5-di(hetero)arylthiophenes were synthesized in moderate to good yields. A single Pd/Cu-catalyst system, without further catalyst addition, and easily available, stable starting materials were used, resulting in a concise and highly efficient route for the synthesis of the title compounds. This novel pseudo five-component synthesis starting from iodo(hetero)arenes is particularly suitable as a direct access to well-defined thiophene oligomers, which are of peculiar interest in materials science.

## Introduction

Over the past decades 2,5-di(hetero)aryl substituted thiophenes [1–2] have constantly attracted a lot of interest, especially as charge-transport materials in electronic [3] and optoelectronic [4–6] devices, but also in drug design as antitumor [7] or anti-inflammatory agents [8] or in plaque imaging [9]. Most commonly the methodological access to these targets has been based upon Pd- or Ni-catalyzed coupling of dihalo thiophenes with organometallic (hetero)aryl derivatives by virtue of Suzuki [10] or Stille [11] coupling. Even though this strategy for the synthesis of symmetrical 2,5-diarylated thiophenes has proven to be efficient and general, all of these synthetic routes share the drawback of ultimately requiring two different halogenated (hetero)arenes and the separate conversion into an organometallic derivative in an additional step. From a practical point of view halogen–metal exchange, transmetalation and isolation occasionally turns out to be tedious and in many cases the use of polar functionality in the substrate is considerably restricted.

In recent years interesting examples of palladium-catalyzed direct C–H activation and arylation of (hetero)aromatics have been reported [12–13]. Although these procedures only employ a single halogenated substrate and avoid the stoichiometric formation of organometallic intermediates the substrate scope is limited to activated heteroaromatic C–H bonds. In addition, sophisticated catalyst systems must be applied, and the efficiency is also variable.

**Scheme 1:**
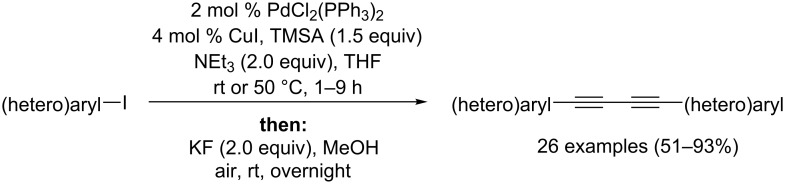
Concept of a Sonogashira–Glaser coupling sequence.

Just recently we reported a very straightforward one-pot synthesis of symmetric 1,4-di(hetero)arylated 1,3-butadiynes starting from (hetero)aryl iodides by virtue of a sequentially Pd/Cu-catalyzed [14] Sonogashira–Glaser process (Scheme 1) [15].

According to this general one-pot access to 1,4-di(hetero)aryl-1,3-butadiynes we reasoned that it should be possible to address the butadiyne functionality towards heterocyclization, again in a one-pot fashion. Here, we communicate the first pseudo five-component synthesis of 2,5-di(hetero)arylthiophenes by virtue of a one-pot Sonogashira–Glaser cyclization sequence.

## Results and Discussion

**Scheme 2:**
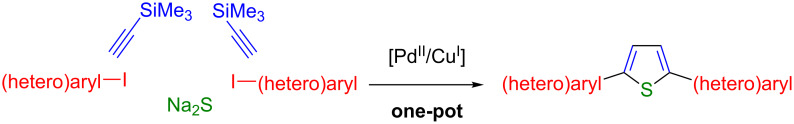
Concept of a Sonogashira–Glaser cyclization synthesis of 2,5-di(hetero)arylthiophenes.

The conversion of 1,4-diaryl-1,3-butadiynes into 2,5-diarylthiophenes by base-mediated cyclization with sodium sulfide or sodium hydrogen sulfide is a literature-known procedure [16–23]. Therefore, we reasoned that the concatenation of our sequentially Pd/Cu-catalyzed Sonogashira–Glaser reaction [15] with the sulfide-mediated cyclization should lead to a straightforward one-pot pseudo five-component synthesis of 2,5-di(hetero)arylthiophenes (Scheme 2).

We first set out to identify an optimal cosolvent for all four steps taking advantage of the high yield Sonogashira–Glaser coupling synthesis [15] of 1,4-diphenylbutadiyne starting from iodobenzene (**1a**) (Table 1). In addition, the final cyclization step to give 2,5-diphenylthiophene (**2a**) was performed under microwave heating at 120 °C for a hold time of 2 h.

**Table 1:** Evaluation of different solvents.^a^

entry	solvent	cavity temperature [°C] (hold time in the cyclization step)	conversion^b^ (yield of **2a** [%]^c^)

1	THF	120 (2 h)	complete (61)
2	1,4-dioxane	120 (2 h)	complete (59)
3	DMSO	120 (2 h)	complete (11)
4	DMF	120 (2 h)	complete (64)
5	DMF	90 (4 h)	complete (n. i.)^d^
6^e^	DMF	90 (8 h)	complete (n. i.)^d^

^a^Reaction conditions: Iodobenzene (2 mmol) in degassed solvent (10 mL) was reacted for 1.5 h at rt with TMSA (3 mmol) in the presence of PdCl_2_(PPh_3_)_2_ (0.04 mmol), CuI (0.08 mmol), and NEt_3_ (2 mmol). Then KF (3 mmol) and methanol (5 mL) were added and the reaction mixture was stirred in the open reaction vessel at rt for 16 h. After the addition of Na_2_S·9H_2_O (3 mmol) and KOH (3 mmol) the sealed reaction vessel was heated in a microwave oven. ^b^Conversion in the final step (monitored by TLC). ^c^Given yields refer to isolated and purified products. ^d^n. i.: Not isolated. ^e^The final step was performed in an oil bath at 90 °C for 8 h to achieve complete conversion.

The solvent screening revealed that THF (tetrahydrofuran) (Table 1, entry 1), 1,4-dioxane (Table 1, entry 2), and DMF (*N*,*N*-dimethylformamide) (Table 1, entry 4) are equally suitable solvents giving rise to essentially comparable yields. DMSO (dimethylsulfoxide) (Table 1, entry 3), however, turned out to give inferior yields, resulting in an increased formation of byproducts already during the desilylation and the oxidative coupling step (as monitored by TLC). A lower reaction temperature resulted in a prolonged reaction time under microwave conditions to achieve complete conversion (Table 1, entry 5), whereas conductive heating at the same temperature even doubled this reaction time (Table 1, entry 6). As a consequence, DMF as a solvent and dielectric heating at 120 °C for 2 h in the final step were identified as the optimal settings for the sequence.

**Scheme 3:**
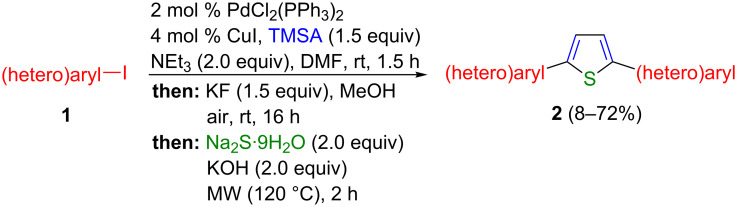
Pseudo five-component Sonogashira–Glaser cyclization synthesis of symmetrical 2,5-di(hetero)arylthiophenes **2**.

**Figure 1:**
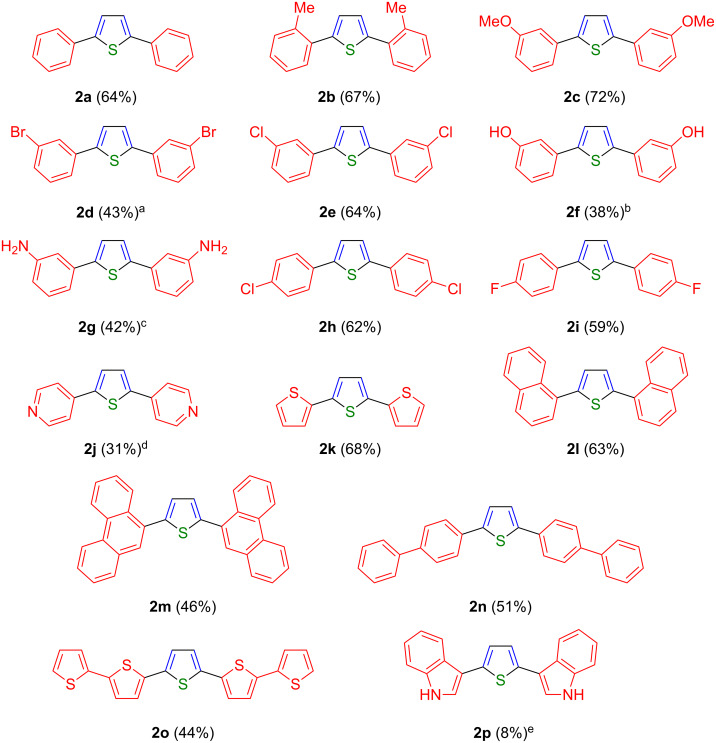
Symmetrical 2,5-di(hetero)arylthiophenes **2** synthesized via the one-pot pseudo five-component Sonogashira–Glaser cyclization sequence (yields refer to 0.5 equiv of (hetero)aryl iodide). ^a^Only one equiv of TMSA was applied in the Sonogashira step. ^b^According to elemental analysis compound **2f** was obtained with 25% hydrate. ^c^*m*-Iodo nitrobenzene (**1g**) was applied as a starting material. ^d^According to elemental analysis, compound **2j** was obtained as a bishydrochloride. ^e^*N*-Boc 3-iodo indole (**1p**) was applied as a starting material.

With these optimized conditions in hand, the substrate scope of this novel pseudo five-component synthesis of 2,5-di(hetero)arylthiophenes was studied (Scheme 3). Starting from (hetero)aryl iodide **1** all reactions were carried out on a 2 mmol scale to give symmetrical 2,5-di(hetero)arylthiophenes **2** as stable, crystalline solids (with the exception of **2b**) in moderate to good yield (Figure 1). The structural assignments of all thiophenes **2** were unambiguously supported by ^1^H and ^13^C NMR spectroscopy, mass spectrometry, and combustion analysis. Due to poor solubility no NMR spectra of compounds **2m**, **2n** and **2o** could be recorded, yet, the assignment of the molecular structure is supported by mass spectrometry and combustion analysis.

The scope of this new one-pot pseudo five-component Sonogashira–Glaser cyclization synthesis of symmetrical 2,5-di(hetero)arylthiophenes **2** is fairly broad with respect to the applied (hetero)aryl iodides **1**. The product analysis of the target structures **2** reveals that aryl substituents can be electroneutral (**2a** and **2l**–**2n**), electron-rich (**2b**, **2c**, **2f**, **2k**, **2o**, **2p**) as well as electron-poor (**2d**, **2e** and **2h**–**2j**). Substituents in *ortho*- (**2b**), *meta*- (**2c**–**2g**,) and *para*-positions (**2h**, **2i**) are tolerated. Even bulky bi- or tricyclic substrates are transformed without any complications (**2l–2p**). Polar substituents such as hydroxy groups (**2f**) are tolerated as well. Furthermore, several different 5- and 6-membered S- and N-heteroaryl iodides give rise to the formation of the corresponding 2,5-di(heteroaryl)thiophenes (**2j–2k** and **2o**) in good yields.

Deviating from the general procedure, in the case of *m*-bromo-iodobenzene (**1d**) only 1 equiv of TMSA was added in order to minimize a second alkynylation at the bromine position in the initial Sonogashira coupling step, which resulted in a moderate yield of the dibromo derivative (**2d**). Upon reaction of the *m*-iodo-nitrobenzene (**1g**) a concomitant reduction of the nitro groups to the amines was observed, giving rise to the dianilino thiophene **2g**.

Most interestingly, even the linear five-ring-containing derivatives “PPTPP” (**2n**) and “T5” (**2o**), which are important charge-transport molecules in materials science [3], were easily accessed in a one-pot procedure. Starting from the stable and readily available aryliodides **1n** and **1o**, the presented new methodology allowed the synthesis of both molecules in a quick, simple and economic one-pot reaction. Moreover, the usual preparation and isolation of boronic acids or even more sensitive zinc organometallics was circumvented. In addition the use of the rather expensive diiodothiophene as a coupling partner was avoided [24–26]. “PPTPP” (**2n**) and “T5” (**2o**) were readily purified by Soxhlet extraction.

Upon reaction of *N*-Boc-3-iodoindole (**1p**) a complete cleavage of the protection group and the formation of several byproducts were observed leading to a significantly lower isolated yield of the corresponding thiophene **2p**.

## Conclusion

In summary we have developed an economical and efficient one-pot sequence for transforming (hetero)aryl iodides into symmetrical 2,5-di(hetero)arylthiophenes based upon an initial sequentially Pd/Cu-catalyzed Sonogashira–Glaser process followed by a subsequent sulfide-mediated cyclization. A broad range of functional groups is tolerated and the iodo substrates are either commercially available or easily accessible. This strikingly simple methodology is highly practical and leads to a straightforward protocol for the preparation of the title compounds. Studies addressing more-sophisticated 2,5-disubstituted thiophenes for surface modification and also mesoporous hybrid materials are currently underway.

## Experimental

**2c:** An 80 mL microwave reaction vessel, equipped with a rubber septum, was charged with 1-iodo-3-methoxybenzene (**1c**) (468 mg, 2.00 mmol), PdCl_2_(PPh_3_)_2_ (28 mg, 0.04 mmol, 2 mol %), CuI (16 mg, 0.08 mmol, 4 mol %), and degassed DMF (10.0 mL). The reaction mixture was flushed for 10 min with nitrogen by using a cannula. After addition of trimethylsilylacetylene (0.43 mL, 3.00 mmol) and dry triethylamine (0.55 mL, 4.00 mmol) the solution was stirred at rt for 1.5 h. Then KF (174 mg, 3.00 mmol), and methanol (5.00 mL) were subsequently added and the reaction mixture was stirred under aerobic atmosphere in the opened reaction vessel overnight at rt. After the addition of sodium sulfide nonahydrate (960 mg, 4 mmol), potassium hydroxide (224 mg, 4 mmol), and methanol (5 mL) the vessel was heated to 120 °C under microwave irradiation for 2 h. After cooling to rt the mixture was adsorbed on neutral aluminium oxide and filtered through a short plug of neutral aluminium oxide with THF as an eluent. The solvents were removed in vacuo and the residue was adsorbed on Celite^®^ and purified by column chromatography on silica gel (hexane) to give 215 mg (0.72 mmol, 72 %) of **2c** as a light-yellow solid. *R*_f_ 0.35 (*n*-hexane/ethyl acetate 10:1); mp 73 °C; ^1^H NMR (CDCl_3_, 500 MHz) δ 3.87 (s, 6H), 6.83–6.87 (m, 2H), 7.16–7.18 (m, 2H), 7.22–7.25 (m, 2H), 7.29 (s, 2H), 7.31 (t, ^3^*J* = 7.9 Hz, 2H); ^13^C NMR (CDCl_3_, 125 MHz) δ 55.5 (CH_3_), 111.4 (CH), 113.2 (CH), 118.4 (CH), 124.3 (CH), 130.1 (CH), 135.7 (C_quat_), 143.6 (C_quat_), 160.1 (C_quat_); EIMS *m*/*z* (%): 297 (22), 296 ([M]^+^, 100), 253 (27), 210 (16), 148 (15); UV–vis (CH_2_Cl_2_), λ_max_ [nm] (ε): 331 (36700); IR (KBr), 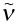 (cm^−1^): 3008 (w), 2960 (w), 2924 (w), 2852 (w), 2833 (w), 1776 (w), 1593 (m), 1581 (m), 1473 (m), 1458 (m), 1436 (m), 1423 (m), 1334 (w), 1319 (m), 1286 (m), 1255 (m), 1197 (m), 1176 (m), 1159 (m), 1120 (m), 1033 (s), 975 (m), 839 (m), 804 (s), 786 (s), 775 (s), 723 (m), 678 (s), 624 (m); Anal. calcd for C_18_H_16_O_2_S (296.4): C, 72.94; H, 5.44; found: C, 73.10; H 5.73.

## Supporting Information

File 1 Experimental procedures, spectroscopic and analytical data of all compounds **2**.

File 2 Copies of NMR spectra of compounds **2a**–**l** and **2p**.
