# Supplementary material for: Pseudo five-component synthesis of 2,5-di(hetero)arylthiophenes via a one-pot Sonogashira–Glaser cyclization sequence
Source: Beilstein J Org Chem. 2011 Nov 4;7:1499–503. doi: 10.3762/bjoc.7.174 (PMC3252849; doi:10.3762/bjoc.7.174)
Supplement: File 1 — Experimental procedures, spectroscopic and analytical data of all compounds 2. [file Beilstein_J_Org_Chem-07-1499-s001.pdf]

Supporting Information

for

Pseudo five-component synthesis of 2,5-di(hetero)arylthiophenes via a one-pot Sonogashira–Glaser cyclization sequence

Dominik Urselmann, Dragutin Antovic, and Thomas J. J. Müller*

Address: Institut für Organische Chemie und Makromolekulare Chemie, Heinrich-Heine-Universität Düsseldorf, Universitätsstr. 1, D-40225 Düsseldorf, Germany.

Email: Dominik Urselmann - Dominik.Urselmann@uni-duesseldorf.de; Dragutin Antovic - Dragutin.Antovic@uni-duesseldorf.de; Thomas J. J. Müller* - ThomasJJ.Mueller@uni-duesseldorf.de

* Corresponding author

Experimental procedures, spectroscopic and analytical data of all compounds 2.

Table of contents

1. General considerations S3
2. Preparation of starting materials S4
2.1. General procedure (GP1) S4
2.1.1. 1-iodonaphthalene (**1l**) S4
2.1.2. 9-iodophenanthrene (**1m**) S4
2.1.3. 4-iodobiphenyl (**1n**) S5
2.1.4. 5-iodo-2,2'-bithiophene (**1o**) S5
3. Sonogashira–Glaser cyclization sequence S6
3.1. General procedure (GP2) S6
3.2. Spectroscopic data of compounds **2a–2p** S7
3.2.1. 2,5-diphenylthiophene (**2a**) S7
3.2.2. 2,5-di(*o*-tolyl)thiophene (**2b**) S8
3.2.3. 2,5-bis(3-methoxyphenyl)thiophene (**2c**) S9
3.2.4. 2,5-bis(3-bromophenyl)thiophene (**2d**) S10
3.2.5. 2,5-bis(3-chlorophenyl)thiophene (**2e**) S11
3.2.6. 3,3'-(thiophene-2,5-diyl)diphenol (**2f**) S12
3.2.7. 3,3'-(thiophene-2,5-diyl)dianiline (**2g**) S13
3.2.8. 2,5-bis(4-chlorophenyl)thiophene (**2h**) S14
3.2.9. 2,5-bis(4-fluorophenyl)thiophene (**2i**) S15
3.2.10. 2,5-bis(pyridin-4-yl)thiophene (**2j**) S16
3.2.11. [2,2’:5’,2’’]-terthiophene (**2k**) S17
3.2.12. 2,5-bis(naphthalen-1-yl)thiophene (**2l**) S18
3.2.13. 2,5-bis(phenanthren-9-yl)thiophene (**2m**) S19
3.2.14. 2,5-bis(biphenyl-4-yl)thiophene (**2n**) S20
3.2.15. [2,2’:5’,2’’:5’’,2’’’:5’’’,2’’’’]-quinquethiophene (**2o**) S21
3.2.16. 2,5-bis(1*H*-indol-3-yl)thiophene (**2p**) S22
4. References S23

1. General considerations

All cross coupling reactions were carried out in oven-dried 80 mL microwave vessels (CEM Corporation) by using septa and syringes under nitrogen atmosphere. THF was dried in an MBraun system MB-SPS-800, and triethylamine was refluxed under argon over ketyl sodium, distilled and stored in a Schlenk flask under nitrogen atmosphere.

5-bromo-2,2'-bisthiophene (**3o**) was prepared according to literature procedures from thiophene [1]. Commercial-grade reagents were used as supplied without further purification and were purchased from Sigma-Aldrich Chemie GmbH, ABCR GmbH & Co. KG, Alfa Aesar GmbH & Co. KG, and Merck Serono KGaA.

The purification of products was performed on silica gel 60 (0.015–0.040 mm) from Macherey-Nagel Düren by using the flash technique under a pressure of 2 bar. The crude mixtures were adsorbed on Celite® 545 (0.02–0.10 mm) from Merck Serono KGaA Darmstadt before chromatographic purification.

The reaction progress was monitored qualitatively by TLC silica gel 60 F_{254} aluminium sheets obtained by Merck Serono KGaA Darmstadt. The spots were detected with UV light at 254 nm and by using aqueous potassium permanganate solution.

^{1}H, ^{13}C, and 135-DEPT NMR spectra were recorded on a Bruker DRX 500 spectrometer. $CDCl_3$, acetone-d_6 and DMSO-d_6 were used as deuterated solvents. The resonances of the residues of nondeuterated solvent were locked as internal standards ($CDCl_3$: ^{1}H δ 7.26, ^{13}C δ 77.2; acetone-d_6: ^{1}H δ 2.05, ^{13}C δ *29.8*, δ *206.3;* DMSO-d_6: ^{1}H δ 2.50, ^{13}C δ *39.5*). The multiplicities of signals were abbreviated as follows: s: singlet; d: doublet; t: triplet; dd: doublet of doublets and m: multiplet. The type of carbon atoms was determined on the basis of 135-DEPT NMR spectra. EI mass spectra were measured on Finnigan MAT 8200 or Shimadzu GC-2010/QP-2010 spectrometer. IR spectra were obtained on Bruker Vector 22 FT-IR. The solids were measured as KBr pellets and oils as films on KBr plates. The intensity of signals is abbreviated as follows: s (strong), m (medium) and w (weak). The melting points (uncorrected) were measured on a Büchi Melting Point B-540. Combustion analyses were carried out on Perkin Elmer Series II Analyser 2400 in the microanalytical laboratory of the Institut für Pharmazeutische und Medizinische Chemie der Heinrich-Heine-Universität Düsseldorf.

2. Preparation of starting materials

2.1. General procedure (GP1)

5 mmol of the bromo(hetero)arene **3** was placed in a dried Schlenk tube and dissolved in 5 mL degassed 1,4-dioxane. After addition of CuI (5 mol %), *N*,*N*'-dimethylethane-1,2-diamine (10 mol %) and NaI (10 mmol) the reaction mixture was heated to 105 °C for 24–72 h. The reaction was monitored by GC–MS. After complete conversion was reached the reaction mixture was absorbed on Celite® and purified by column chromatography on silica gel with *n*-hexane.

2.1.1. 1-iodonaphthalene (1l)

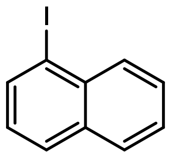

$C_{10}H_7I$

254.07

The synthesis was carried out according to the general procedure **GP1** with 1035 mg (5.00 mmol) 1-bromonaphthalene (**3l**). The crude product was purified by column chromatography with *n*-hexane as an eluant to give 1242 mg (4.89 mmol, 98%) of the desired product as a colorless liquid.

MS (EI$^+$) *m*/*z* (%): 254 ([M]$^+$, 85), 128 (11), 127 (100), 126 (21), 77 (13), 75 (11).

2.1.2. 9-iodophenanthrene (1m)

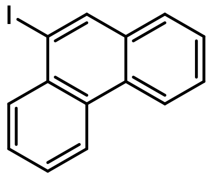

$C_{14}H_9I$

304.13

The synthesis was carried out according to the general procedure **GP1** with 1286 mg (5.00 mmol) 9-bromophenanthrene (**3m**). The crude product was purified by column

chromatography with *n*-hexane as an eluant to give 700 mg (2.30 mmol, 46%) of the desired product as a light-yellow solid.

MS (EI^+) *m/z* (%): 305 (15), 304 ($[M]^+$, 100), 178 (11), 177 (75), 176 (56), 152 (24), 151 (17), 150 (16), 88 (59), 75 (17).

2.1.3. 4-iodobiphenyl (1n)

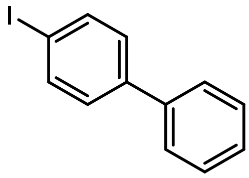

$C_{12}H_9I$

280.10

The synthesis was carried out according to the general procedure **GP1** with 1166 mg (5.00 mmol) 4-bromobiphenyl (**3n**). The crude product was purified by column chromatography with *n*-hexane as an eluant to give 1240 mg (4.42 mmol, 89%) of the desired product as a colorless solid.

MS (EI^+) *m/z* (%): 281 (12), 280 ($[M]^+$, 100), 153 (28), 152 (73), 151 (19), 76 (33), 75 (10), 63 (11).

2.1.4. 5-iodo-2,2'-bithiophene (1o)

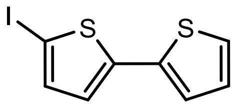

$C_8H_5IS_2$

292.16

The synthesis was carried out according to the general procedure **GP1** with 1226 mg (5.00 mmol) 5-bromo-2,2'-bithiophene (**3o**). The crude product was purified by column chromatography with *n*-hexane as an eluant to give 1312 mg (4.49 mmol, 90%) of the desired product as a colorless solid.

MS (EI^+) *m/z* (%): 293 (11), 292 ($[M]^+$, 100), 165 (23), 121 (89), 82 (20), 77 (12), 69 (18), 63 (11).

3. Sonogashira–Glaser cyclization sequence

3.1. General procedure (GP2)

A mixture of the (hetero)aryl halide **1** (2.00 mmol), $PdCl_2(PPh_3)_2$ (28 mg, 0.04 mmol, 2 mol %) and CuI (16 mg, 0.08 mmol, 4 mol %) was dissolved in degassed DMF (10.0 mL) in a 80 mL microwave vessel equipped with a stirring bar and a septum. After addition of trimethylsilylacetylene (0.43 mL, 3.00 mmol) and dry triethylamine (0.55 mL, 4.00 mmol) the solution was stirred at rt for 1.5 h (conversion was monitored by TLC). Then KF (174 mg, 3.00 mmol) and methanol (5.00 mL) were added subsequently and the reaction mixture was stirred in air in the open reaction vessel overnight at rt (conversion was monitored by TLC). After addition of sodium sulfide nonahydrate (960 mg, 4 mmol), potassium hydroxide (224 mg, 4 mmol) and methanol (5 mL), the mixture was heated to 120 °C for 2 h by microwave irradiation. After cooling to rt the mixture was absorbed on neutral aluminium oxide and filtered through neutral aluminium oxide by using THF. The resulting solution was treated as given below.

3.2. Spectroscopic data of compounds 2a–2p

3.2.1. 2,5-diphenylthiophene (2a)

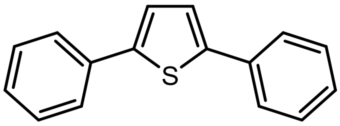

$C_{16}H_{12}S$

236.33

The synthesis was carried out according to the general procedure **GP2** with 418 mg (2.00 mmol) of iodobenzene (**1a**) (Alfa Aesar). The crude product was absorbed onto Celite® and purified by column chromatography on silica gel with *n*-hexane as an eluant to give 151 mg (0.64 mmol, 64%) of the desired product as colorless crystals.

R_f 0.29 (*n*-hexane); mp 153 °C; ^{1}H NMR ($CDCl_3$, 500 MHz) δ 7.28–7.33 (m, 4H), 7.38–7.44 (m, 4H), 7.63–7.69 (m, 4H); ^{13}C NMR ($CDCl_3$, 125 MHz) δ 124.1 (CH), 125.8 (CH), 127.6 (CH), 129.1 (CH), 134.4 (C_{quat}), 143.7 (C_{quat}); MS (EI^+) *m*/*z* (%): 238 (12), 237 (38), 236 ($[M]^+$, 100), 234 (16), 202 (15), 134 (11), 121 (31), 118 (25), 115 (13), 77 (12); UV–vis (CH_2Cl_2), λ_{max} [nm] (ε): 326 (49800); IR (KBr), $\tilde{\nu}$ [cm^{-1}]: 3074 (w), 3055 (w), 3018 (w), 2958 (w), 2922 (w) , 2220 (w), 1882 (w), 1595 (w), 1479 (m), 1454 (m), 1415 (w), 1327 (w), 1273 (w), 1205 (w), 1182 (m), 1155 (m), 1078 (m), 1051 (m), 1028 (m), 950 (m), 939 (m), 902 (m), 839 (m), 804 (m), 746 (s), 684 (s), 605 (m); EA calcd for $C_{16}H_{12}S$ (236.3): C, 81.31; H, 5.12; found: C, 81.15; H 5.24.

3.2.2. 2,5-di(*o*-tolyl)thiophene (2b)

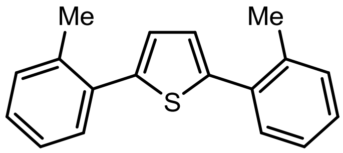

$C_{18}H_{16}S$

264.38

The synthesis was carried out according to the general procedure **GP2** with 436 mg (2.00 mmol) of 1-iodo-2-methylbenzene (**1b**) (Merck). The crude product was absorbed onto Celite® and purified by column chromatography on silica gel with *n*-hexane as an eluant to give 178 mg (0.67 mmol, 67%) of the desired product as a colorless liquid.

R_f 0.36 (*n*-hexane); ^{1}H NMR ($CDCl_3$, 500 MHz) δ 2.51 (s, 6H), 7.06 (s, 2H), 7.22–7.31 (m, 6H), 7.46–7.50 (m, 2H); ^{13}C NMR ($CDCl_3$, 125 MHz) δ 21.5 (CH_3), 126.1 (CH), 126.6 (CH), 127.9 (CH), 130.4 (CH), 131.0 (CH) 134.2 (C_{quat}), 136.1 (C_{quat}), 143.0 (C_{quat}); MS (EI^+) *m*/*z* (%): 265 (21), 264 ($[M]^+$, 100), 216 (15), 215 (17), 149 (10), 148 (71), 147 (39), 135 (25), 116 (10), 115 (35), 91 (12); UV–vis (CH_2Cl_2), λ_{max} [nm] (ε): 295 (23200); IR (KBr), $\tilde{\nu}$ [cm^{-1}]: 3061 (m), 3015 (m), 2952 (m), 2923 (m), 1914 (w), 1600 (m), 1482 (s), 1456 (s), 1379 (m), 1379 (m), 1256 (m), 1205 (m), 1160 (w), 1120 (m), 1034 (m), 969 (m), 933 (m), 811 (m), 756 (s), 722 (s), 666 (m), 588 (m), 531 (m); EA calcd for $C_{18}H_{16}S$ (264.4): C, 81.77; H, 6.10; found: C, 81.54; H, 5.93.

3.2.3. 2,5-bis(3-methoxyphenyl)thiophene (2c)

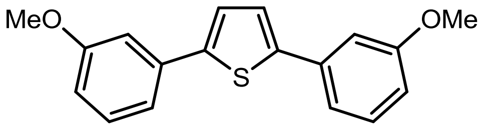

$C_{18}H_{16}O_2S$

296.38

The synthesis was carried out according to the general procedure **GP2** with 468 mg (2.00 mmol) of 1-iodo-3-methoxybenzene (**1c**) (ABCR). The crude product was absorbed onto Celite® and purified by column chromatography on silica gel with *n*-hexane:ethyl acetate 10/1 as an eluant to give 215 mg (0.72 mmol, 72%) of the desired product as a light-yellow solid.

R_f 0.35 (*n*-hexane:ethyl acetate 10/1); mp 73 °C; ^{1}H NMR ($CDCl_3$, 500 MHz) δ 3.87 (s, 6H), 6.83–6.87 (m, 2H), 7.16–7.18 (m, 2H), 7.22–7.25 (m, 2H), 7.29 (s, 2H), 7.31 (t, ^3J = 7.9 Hz, 2H); ^{13}C NMR ($CDCl_3$, 125 MHz) δ 55.5 (CH_3), 111.4 (CH), 113.2 (CH), 118.4 (CH), 124.3 (CH), 130.1 (CH), 135.7 (C_{quat}), 143.6 (C_{quat}), 160.1 (C_{quat}); MS (EI^+) *m*/*z* (%): 297 (22), 296 ($[M]^+$, 100), 253 (27), 210 (16), 148 (15); UV–vis (CH_2Cl_2), λ_{max} [nm] (ε): 331 (36700); IR (KBr), $\tilde{\nu}$ [cm^{-1}]: 3008 (w), 2960 (w), 2924 (w), 2852 (w), 2833 (w), 1776 (w), 1593 (m), 1581 (m), 1473 (m), 1458 (m), 1436 (m), 1423 (m), 1334 (w), 1319 (m), 1286 (m), 1255 (m), 1197 (m), 1176 (m), 1159 (m), 1120 (m), 1033 (s), 975 (m), 839 (m), 804 (s), 786 (s), 775 (s), 723 (m), 678 (s), 624 (m); EA calcd for $C_{18}H_{16}O_2S$ (296.4): C, 72.94; H, 5.44; found: C, 73.10; H, 5.73.

3.2.4. 2,5-bis(3-bromophenyl)thiophene (2d)

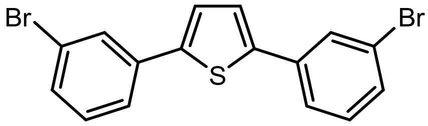

$C_{16}H_{10}Br_2S$

394.12

The synthesis was carried out according to the general procedure **GP2** with 566 mg (2.00 mmol) of 1-bromo-3-iodobenzene (**1d**) (ABCR). As an exception to the general procedure **GP2**, only 2 mmol of trimethylsilylacetylene was used. The crude product was absorbed onto Celite® and purified by column chromatography on silica gel with *n*-hexane:ethyl acetate 10/1 as an eluant to give 169 mg (0.43 mmol, 43%) of the desired product as a light-yellow solid.

R_f 0.39 (*n*-hexane); mp 181 °C (decomp); ^{1}H NMR ($CDCl_3$, 500 MHz) δ 7.26 (t, ^3J = 7.9 Hz, 2H), 7.29 (s, 2H), 7.42 (ddd, ^3J = 7.9 Hz, ^4J = 1.8 Hz, ^4J = 1.0 Hz, 2H), 7.54 (ddd, ^3J = 7.8 Hz, ^4J = 1.7 Hz, ^4J = 1.0 Hz, 2H), 7.77 (t, ^4J = 1.8 Hz, 2H); ^{13}C NMR ($CDCl_3$, 125 MHz) δ 123.2 (C_{quat}), 124.4 (CH), 125.0 (CH), 128.7 (CH), 130.6 (CH), 130.7 (CH), 136.2 (C_{quat}), 142.7 (C_{quat}); MS (EI$^+$) *m*/*z* (%): 395 (52), 394 (19), 393 ([M]$^+$, 100), 391 (50), 234 (42), 233 (10), 232 (13), 202 (14), 201 (12), 199 (10), 197 (12), 197 (23), 196 (11), 189 (22), 120 (15), 117 (43), 116 (22), 104 (20), 95 (17), 94 (11), 89 (15), 75 (11), 63 (11); UV–vis (CH_2Cl_2), λ_{max} [nm] (ε): 330 (35600); IR (KBr), $\tilde{\nu}$ [cm^{-1}]: 3053 (w),1869 (w), 1581 (w), 1552 (m), 1541 (w), 1458 (m), 1408 (m), 1336 (w), 1255 (w), 1199 (w), 1161 (w), 1056 (m), 1033 (w), 991 (m), 879 (m), 840 (w), 798 (w), 773 (s), 746 (m), 719 (s), 684 (s), 669 (s), 651 (m); EA calcd for $C_{16}H_{12}Br_2S$ (394.1): C, 48.76; H, 2.56; found: C, 48.81; H, 2.43.

3.2.5. 2,5-bis(3-chlorophenyl)thiophene (2e)

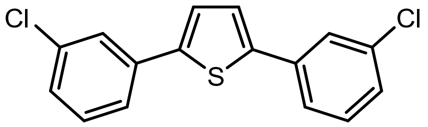

$C_{16}H_{10}Cl_2S$

305.22

The synthesis was carried out according to the general procedure **GP2** with 477 mg (2.00 mmol) of 1-chloro-3-iodobenzene (**1e**) (ABCR). The crude product was absorbed onto Celite® and purified by column chromatography on silica gel with *n*-hexane as an eluant to give 194 mg (0.64 mmol, 64%) of the desired product as colorless crystals.

R_f 0.38 (*n*-hexane); mp 134 °C; ^{1}H NMR ($CDCl_3$, 500 MHz) δ 7.18–7.22 (m, 2H), 7.23 (s, 2H), 7.26 (d, ^3J = 7.8 Hz, 2H), 7.43 (ddd, ^3J = 1.2, ^4J = 1.7, ^4J = 7.7, 2H), 7.54 (t, ^3J = 1.77, 2H); ^{13}C NMR ($CDCl_3$, 125 MHz) δ 124.2 (2 CH), 125.3 (2 CH), 126 (2 CH), 128 (2 CH), 130.6 (2 CH), 135.3 (2 C_{quat}), 136.2 (2 C_{quat}), 143.1 (2 C_{quat}); MS (EI^+) *m*/*z* (%): 308 (14), 307 (12), 306 (69), 305 (18), 304 ($[M]^+$, 100), 234 (17), 155 (23), 153 (13), 152 (20), 116 (12); UV–vis (CH_2Cl_2), λ_{max} [nm] (ε): 329 (44600); IR (KBr), $\tilde{\nu}$ [cm^{-1}]: 2980 (w), 2972 (w), 1589 (w), 1560 (m), 1543 (w), 1458 (m), 1413 (m), 1336 (w), 1257 (w), 1080 (m), 1001 (w), 881 (m), 790 (m), 775 (s), 761 (s), 736 (m), 686 (s), 659 (m); EA calcd for $C_{16}H_{10}Cl_2S$ (305.2): C, 62.96; H, 3.30; found: C, 63.11; H, 3.44.

3.2.6. 3,3'-(thiophene-2,5-diyl)diphenol (2f)

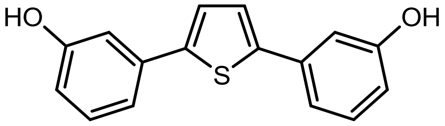

$C_{16}H_{12}O_2S$

268.33

The synthesis was carried out according to the general procedure **GP2** with 440 mg (2.00 mmol) of 3-iodophenol (**1f**) (ABCR). The crude product was absorbed onto Celite® and purified by column chromatography on silica gel with *n*-hexane:ethyl acetate 2/1 as an eluant to give 102 mg (0.37 mmol, 37%) of the desired product as a colorless solid.

R_f 0.28 (*n*-hexane:ethyl acetate 2/1); mp 211 °C; ^{1}H NMR (acetone-d_6, 500 MHz) δ 6.77–6.83 (m, 2H), 7.14–7.20 (m, 4H), 7.21–7.27 (m, 2H), 7.39 (s, 2H), 8.52 (s, 2H); ^{13}C NMR (acetone-d_6, 125 MHz) δ 113.0 (CH), 115.6 (CH), 117.6 (CH), 125.2 (CH), 131.0 (CH), 136.3 (C_{quat}), 144.1 (C_{quat}), 158.8 (C_{quat}); MS (EI$^+$) *m*/*z* (%): 269 (22), 268 (100); EA calcd for $C_{16}H_{12}O_2S \cdot 0.25H_2O$ (268.3+4.50): C, 70.44; H, 4.62; found: C, 70.31; H, 4.70.

3.2.7. 3,3'-(thiophene-2,5-diyl)dianiline (2g)

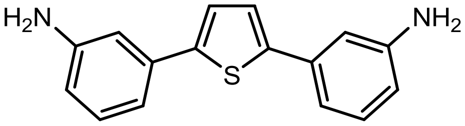

$C_{16}H_{14}N_2S$

266.36

The synthesis was carried out according to the general procedure **GP2** with 498 mg (2.00 mmol) of 1-iodo-3-nitrobenzene (**1g**) (ABCR). The crude product was absorbed onto Celite® and purified by column chromatography on silica gel with *n*-hexane:ethyl acetate 3/2 as an eluant to give 140 mg (0.42 mmol, 42%) of the desired product as a yellow solid.

R_f 0.11 (*n*-hexane:ethyl acetate 3/2); mp 182 °C; ^{1}H NMR (DMSO-d_6, 500 MHz) δ 5.21 (s, 4H), 6.51 (d, ^3J = 7.1 Hz, 2H), 6.79–6.88 (m, 4H), 7.05 (t, ^3J = 7.8 Hz, 2H), 7.31 (s, 2H); ^{13}C NMR (DMSO-d_6, 126 MHz) δ 110.4 (CH), 112.8 (CH), 113.5 (CH), 123.8 (CH), 129.6 (CH), 134.1 (C_{quat}), 142.9 (C_{quat}), 149.2 (C_{quat}); MS (EI^+) *m/z* (%): 267 (20), 266 ($[M]^+$, 100); UV–vis (CH_2Cl_2), λ_{max} [nm] (ε): 336 (30100); EA calcd. for $C_{16}H_{14}N_2S$ (266.4): C 72.15, H 5.30, N 10.52; Found: C 72.00, H 5.57, N 10.45.

3.2.8. 2,5-bis(4-chlorophenyl)thiophene (2h)

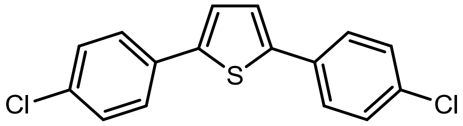

$C_{16}H_{10}Cl_2S$

305.22

The synthesis was carried out according to the general procedure **GP2** with 477 mg (2.00 mmol) of 1-chloro-4-iodobenzene (**1h**) (ABCR). The crude product was absorbed onto Celite® and purified by column chromatography on silica gel with *n*-hexane:ethyl acetate (100:1) as an eluant to give 189 mg (0.62 mmol, 62%) of the desired product as a light-yellow solid.

R_f (*n*-hexane:ethyl acetate = 100:1) = 0.44. mp = 159 °C; ^{1}H NMR ($CDCl_3$, 500 MHz): δ = 7.26 (s, 2H), 7.34–7.38 (m, 4H), 7.52–7.56 (m, 4H); ^{13}C NMR ($CDCl_3$, 125 MHz): δ = 124.6 (CH), 126.9 (CH), 129.3 (CH), 132.8 (C_{quat}), 133.6 (C_{quat}), 142.8 (C_{quat}); MS (EI^+) *m/z* (%): 308 (14), 307 (12), 306 (69), 305 (18), 304 ($[M]^+$, 100), 234 (17), 157 (10), 155 (26), 153 (17), 152 (25), 116 (11); UV–vis (CH_2Cl_2), λ_{max} [nm] (ε): 333 (51100); IR (KBr), $\tilde{\nu}$ [cm^{-1}]: 2833 (w), 2360 (w), 1897 (w), 1593 (w), 1537 (w), 1477 (w), 1448 (w), 1406 (w), 1375 (w), 1336 (w), 1257 (w), 1222 (w), 1161 (w), 1093 (m), 1068 (w), 1033 (w), 1010 (m), 968 (w), 937 (w), 881 (w), 827 (m), 798 (s), 777 (m), 732 (m), 680 (w); EA calcd. for $C_{16}H_{10}Cl_2S$ (305.2): C 62.96, H 3.30; Found: C 63.18, H 3.10.

3.2.9. 2,5-bis(4-fluorophenyl)thiophene (2i)

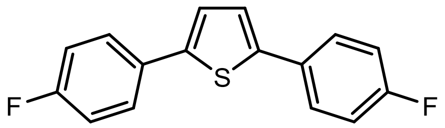

$C_{16}H_{10}F_2S$

272.31

The synthesis was carried out according to the general procedure **GP2** with 444 mg (2.00 mmol) of 1-fluoro-4-iodobenzene (**1i**) (ABCR). The crude product was absorbed onto Celite® and purified by column chromatography on silica gel with *n*-hexane as an eluant to give 161 mg (0.59 mmol, 59%) of the desired product as colorless crystals.

R_f (*n*-hexane) = 0.32; mp = 163 °C; ^{1}H NMR ($CDCl_3$, 500 MHz): δ = 7.05–7.12 (m, 4H), 7.20 (s, 2H), 7.55–7.61 (m, 4H.); ^{13}C NMR ($CDCl_3$, 125 MHz): δ = 116.1 (d, $^2J_{CF}$ = 21.8 Hz, CH), 124.1 (CH), 127.4 (d, $^3J_{CF}$ = 8.0 Hz, CH), 130.6 (d, $^4J_{CF}$ = 3.4 Hz, C_{quat}), 142.7 (C_{quat}), 162.5 (d, $^1J_{CF}$ = 247.5 Hz, C_{quat}); MS (EI^+) m/z (%): 273 (17), 272 ($[M]^+$, 100), 139 (28), 136 (21), 133 (10); UV–vis (CH_2Cl_2), λ_{max} [nm] (ε): 321 (26500); IR (KBr), $\tilde{\nu}$ [cm^{-1}]: 2461 (w), 2220 (w), 1764 (w), 1589 (m), 1541 (m), 1492 (m), 1456 (m), 1404 (m), 1230 (m), 1220 (m), 1186 (m), 1157 (m), 1101 (m), 1074 (m), 1051 (m), 1010 (m), 939 (m), 837 (s), 827 (s), 812 (m), 794 (s), 675 (m), 615 (m); EA calcd. for $C_{16}H_{10}F_2S$ (272.3): C 70.57, H 3.70; Found: C 70.56, H 3.89.

3.2.10. 2,5-bis(pyridin-4-yl)thiophene (2j)

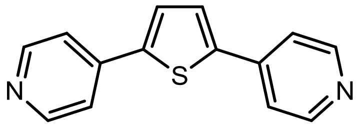

$C_{14}H_{10}N_2S$

238.31

The synthesis was carried out according to the general procedure **GP2** with 410 mg (2.00 mmol) of 4-iodopyridine (**1j**) (ABCR). The crude product was absorbed onto Celite® and purified by column chromatography on silica gel with *n*-hexane:ethyl acetate (3:2) as an eluant. The crude product was dissolved in chloroform. After the addition of a small amount of hydrochloric acid a light-brown precipitate was formed, which was isolated by filtration and dried under reduced pressure to give 97 mg (0.40 mmol, 31%) of the desired product as a yellow solid.

R_f (*n*-hexane:ethyl acetate = 3:2) = 0.25; mp = 162 °C; ^{1}H NMR ($CDCl_3$, 500 MHz): δ = 7.50 (dd, ^3J = 4.6 Hz, ^4J = 1.6 Hz, 4H), 7.52 (s, 2H), 8.63 (d, ^3J = 5.5 Hz, 4H); ^{13}C NMR ($CDCl_3$, 125 MHz): δ = 119.9 (CH), 126.7 (CH), 140.8 (C_{quat}), 142.7 (C_{quat}), 150.6 (CH); MS (EI^+) m/z (%): 239 (17), 238 ($[M]^+$, 100); EA calcd. for $C_{14}H_{10}N_2S \cdot 2HCl$ (311.2): C 54.03, H 3.89, N 9.00; Found: C 54.02, H 4.34, N 8.72.

3.2.11. [2,2':5',2'']-terthiophene (2k)

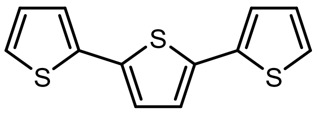

$C_{12}H_8S_3$

248.39

The synthesis was carried out according to the general procedure **GP2** with 420 mg (2.00 mmol) of 2-iodothiophene (**1k**) (Aldrich). The crude product was absorbed onto Celite® and purified by column chromatography on silica gel with *n*-hexane as an eluant to give 165 mg (0.66 mmol, 66%) of the desired product as a light-yellow solid.

R_f (*n*-hexane) = 0.32; mp = 93 °C; ^{1}H NMR ($CDCl_3$, 500 MHz): δ = 7.01–7.05 (m, 2H), 7.08 (s, 2H), 7.18 (d, ^3J = 3.6 Hz, 2H), 7.22 (d, ^3J = 5.1 Hz, 2H); ^{13}C NMR ($CDCl_3$, 125 MHz): δ = 123.8 (CH), 124.4 (CH), 124.6 (CH), 128.0 (CH), 136.3 (C_{quat}), 137.3 (C_{quat}); MS (EI$^+$) *m/z* (%): 248 ([M]$^+$, 100), 203 (12), 124 (12); UV–vis (CH_2Cl_2), λ_{max} [nm] (ε): 353 (59300); IR (KBr), $\tilde{\nu}$ [cm^{-1}]: 2956 (w), 2920 (w), 2353 (w), 2220 (w), 1608 (w), 1494 (m), 1421 (m), 1373 (w), 1346 (w), 1230 (m), 1157 (w), 1122 (w), 1051 (m), 950 (m), 881 (w), 831 (m), 794 (s), 769 (m), 740 (m), 684 (s), 673 (s); EA calcd. for $C_{12}H_8S_3$ (248.4): C 58.03, H 3.25; Found: C 57.83, H 3.32.

3.2.12. 2,5-bis(naphthalen-1-yl)thiophene (2l)

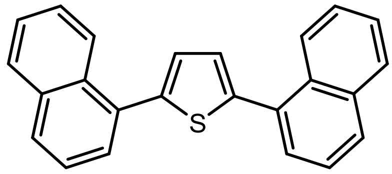

$C_{24}H_{16}S$

336.45

The synthesis was carried out according to the general procedure **GP2** with 508 mg (2.00 mmol) of 1-iodonaphthalene (**1l**). The crude product was absorbed onto Celite® and purified by column chromatography on silica gel with *n*-hexane as an eluant to give 212 mg (0.63 mmol, 63%) of the desired product as a colorless solid.

R_f (*n*-hexane) = 0.38; mp = 140 °C; ^{1}H NMR (acetone-d_6, 500 MHz) δ = 7.44 (s, 2H), 7.57–7.65 (m, 6H), 7.69–7.76 (m, 2H), 7.96-8.05 (m, 4H), 8.38–8.44 (m, 2H); ^{13}C NMR (acetone-d_6, 125 MHz): δ = 126.3 (CH), 126.3 (CH), 127.1 (CH), 127.6 (CH), 128.9 (CH), 129.0 (CH), 129.4 (CH), 129.5 (CH), 132.5 (C_{quat}), 132.9 (C_{quat}), 135.1 (C_{quat}), 142.8 (C_{quat}); MS (EI$^+$) *m/z* (%): 337 (19), 336 ([M]$^+$, 68); UV–vis (CH_2Cl_2) λ_{max} [nm] (ε): 326 (20700); IR (KBr), $\tilde{\nu}$ [cm^{-1}]: 3037 (w), 2922 (m), 2852 (m), 1929 (w), 1587 (m), 1506 (m), 1450 (w), 1390 (m), 1284 (w), 1205 (w), 1159 (w), 1101 (w), 1049 (w), 1020 (m), 937 (w), 872 (m), 829 (m), 793 (s), 773 (s), 659 (m), 642 (m); EA calcd. for $C_{24}H_{16}S$ (336.5): C 85.68, H 4.79; Found: C 85.48, H 5.02.

3.2.13. 2,5-bis(phenanthren-9-yl)thiophene (2m)

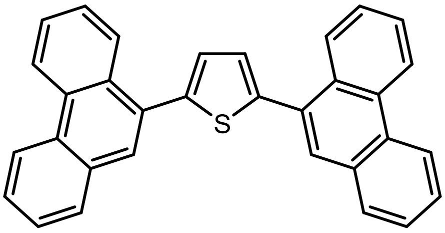

$C_{32}H_{20}S$

436.57

The synthesis was carried out according to the general procedure **GP2** with 608 mg (2.00 mmol) of 9-iodophenanthrene (**1m**). The crude product was absorbed onto Celite® and purified by column chromatography on silica gel with *n*-hexane:ethyl acetate (20:1) as an eluant to give 201 mg (0.46 mmol, 46%) of the desired product as a light-yellow solid.

R_f (*n*-hexane) = 0.22; mp = 189 °C; MS (EI$^+$) *m/z* (%): 438 (11), 437 (35), 436 ([M]$^+$, 100), 233 (10), 221 (11), 218 (14), 200 (13); UV–vis (CH_2Cl_2), λ_{max} [nm] (ε): 253 (119900), 323 (33100); IR (KBr), $\tilde{\nu}$ [cm^{-1}]: 3055 (w), 3034 (w), 2920 (w), 2850 (w), 2715 (w), 1905 (w), 1819 (w), 1676 (w), 1593 (m), 1557 (m), 1541 (w), 1481 (m), 1445 (m), 1408 (m), 1348 (w), 1286 (w), 1217 (m), 1159 (w), 1134 (m), 1072 (w), 1038 (m), 1001 (m), 941 (m), 908 (w), 839 (s), 827 (m), 799 (s), 758 (s), 718 (s), 685 (s); EA calcd. for $C_{32}H_{20}S$ (436.6): C 88.04, H 4.62; Found: C 87.89, H 4.86.

After drying the substance was found to be insoluble in the usual deuterated solvents.

3.2.14. 2,5-bis(biphenyl-4-yl)thiophene (2n)

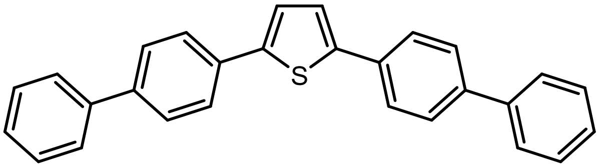

$C_{28}H_{20}S$

388.52

The synthesis was carried out according to the general procedure **GP2** with 560 mg (2.00 mmol) of 4-iodobiphenyl (**1n**). The crude product was absorbed onto aluminium oxide and the resulting solid was washed with 50 mL of tetrahydrofuran and 50 mL of water. The leftover solid was extracted for 16 h with 400 mL of 1,4-dioxane by using a *Soxhlet*-extractor. The solvent was removed under reduced pressure and the residue was recrystallized from 1,4-dioxane to give 198 mg (0.51 mmol, 51%) of the desired product as a yellow solid.

mp = 329 °C; MS (EI^+) *m/z* (%): 389 ([M]+, 30), 388 ($[M]^+$, 100), 194 (36); UV–vis (CH_2Cl_2), λ_{max} [nm] (ε): 355; IR (KBr), $\tilde{\nu}$ [cm^{-1}]: 3057 (w), 3024 (w), 1888 (w), 1819 (w), 1682 (w), 1481 (m), 1447 (m), 1379 (w), 1230 (w), 1196 (w), 1161 (w), 1031 (m), 1001 (w), 941 (w), 891 (m), 837 (w), 815 (m), 769 (s), 741 (s), 721 (s), 684 (w), 617 (m); EA calcd. for $C_{28}H_{20}S$ (388.5): C 86.56, H 5.19; Found: C 86.36, H 4.96.

After drying, the substance was found to be insoluble in the usual deuterated solvents.

3.2.15. [2,2’:5’,2’’:5’’,2’’’:5’’’,2’’’’]-quinquethiophene (2o)

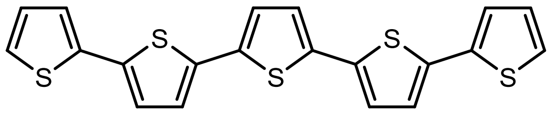

$C_{20}H_{12}S_5$

412.63

The synthesis was carried out according to the general procedure **GP2** with 584 mg (2.00 mmol) of 5-iodo-2,2'-bithiophene (**1o**). The crude product was absorbed onto aluminium oxide and the resulting solid was washed with 50 mL of ethyl acetate and 50 mL of water. The leftover solid was extracted for 16 h with 300 mL of 1,4-dioxane by using a *Soxhlet*-extractor. The solvent was removed under reduced pressure and the residue was recrystallized from 1,4-dioxane to give 185 mg (0.45 mmol, 45%) of the desired product as an orange-brown solid.

mp = 255 °C MS (EI^+) *m/z* (%): 413 (26), 412 ($[M]^+$,100), 206 (20),184 (43), 173 (18); UV–vis (CH_2Cl_2), λ_{max} [nm] (ε): 417 (43200); EA calcd. for $C_{20}H_{12}S_5$ (412.6): C 58.21, H 2.93; Found: C 57.96, H 3.17.

After being dried, the substance was found to be insoluble in the usual deuterated solvents.

3.2.16. 2,5-bis(1*H*-indol-3-yl)thiophene (2p)

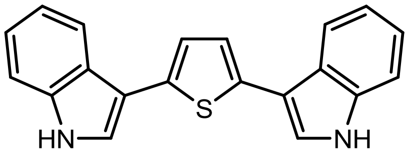

$C_{20}H_{14}N_2S$

314.40

The synthesis was carried out according to the general procedure **GP2** with 598 mg (2.00 mmol) of *tert*-butyl-3-iodo-1*H*-indole-1-carboxylate (**1p**). The crude product was absorbed onto Celite® and purified by column chromatography on silica gel with *n*-hexane:ethyl acetate (2:1) as an eluant to give 24 mg (0.08 mmol, 8%) of the desired product as a light-brown solid.

R_f (*n*-hexane:ethyl acetate = 2:1) = 0.28; mp = 237 °C; ^{1}H NMR (acetone-d_6, 500 MHz): δ = 7.13–7.23 (m, 4H), 7.28–7.36 (m, 2H), 7.44–7.52 (m, 2H), 7.63–7.70 (m, 2H), 7.98–8.05 (m, 2H), 10.50 (s, 2H); ^{13}C NMR (acetone-d_6, 125 MHz): δ = 112.0 (C_{quat}), 112.7 (CH), 120.4 (CH), 120.8 (CH), 122.9 (CH), 123.2 (CH), 123.3 (CH), 126.3 (C_{quat}), 135.8 (C_{quat}), 138.0 (C_{quat}); MS (EI$^+$) *m/z* (%): 315 (24), 314 ([M]$^+$, 100), 313 (17), 157 (22), 94 (10), 91 (16); UV–vis (CH_2Cl_2), λ_{max} [nm] (ε): 283 (13900), 340 (23500); EA calcd. for $C_{20}H_{14}N_2S$ (314.4): C 76.40, H 4.49, N 8.91; Found: C 76.13, H 4.69, N 8.76.

4. References

[1] (a) T. Washino, M. Yoshikura, S. Obata, *Agric. Biol. Chem.* **1986**, *50*, 565–568. (b) Patent US 5591761, „Thiophenyl-, Furyl- and Pyrrolyl-sulfonamides and derivatives thereof that modulate the activity of Endothelin“, M. F. Chan; B. G. Raju; A. Kois; E. J. Verner; C. Wu; R. S. Castillo; V. Yalamoori; V. N. Balaji, **1997**.
