# Supplementary material for: Pseudo five-component synthesis of 2,5-di(hetero)arylthiophenes via a one-pot Sonogashira–Glaser cyclization sequence
Source: Beilstein J Org Chem. 2011 Nov 4;7:1499–503. doi: 10.3762/bjoc.7.174 (PMC3252849; doi:10.3762/bjoc.7.174)
Supplement: File 2 — Copies of NMR spectra of compounds 2a–l and 2p. [file Beilstein_J_Org_Chem-07-1499-s002.pdf]

Supporting Information

for

Pseudo five-component synthesis of 2,5-di(hetero)arylthiophenes via a one-pot Sonogashira–Glaser cyclization sequence

Dominik Urselmann, Dragutin Antovic, and Thomas J. J. Müller*

Address: Institut für Organische Chemie und Makromolekulare Chemie, Heinrich-Heine-Universität Düsseldorf, Universitätsstr. 1, D-40225 Düsseldorf, Germany.

Email: Dominik Urselmann - Dominik.Urselmann@uni-duesseldorf.de; Dragutin Antovic - Dragutin.Antovic@uni-duesseldorf.de; Thomas J. J. Müller* - ThomasJJ.Mueller@uni-duesseldorf.de

* Corresponding author

Copies of NMR spectra of compounds 2a–l and 2p.

Table of contents

2,5-Diphenylthiophene (**2a**) S2
2,5-Di(*o*-tolyl)thiophene (**2b**) S4
2,5-Bis(3-methoxyphenyl)thiophene (**2c**) S6
2,5-Bis(3-bromophenyl)thiophene (**2d**) S8
2,5-Bis(3-chlorophenyl)thiophene (**2e**) S10
3,3'-(Thiophene-2,5-diyl)diphenol (**2f**) S12
3,3'-(Thiophene-2,5-diyl)dianiline (**2g**) S14
2,5-Bis(4-chlorophenyl)thiophene (**2h**) S16
2,5-Bis(4-fluorophenyl)thiophene (**2i**) S18
2,5-Bis(pyridin-4-yl)thiophene (**2j**) S20
[2,2’:5’,2’’]-Terthiophene (**2k**) S22
2,5-Bis(naphthalen-1-yl)thiophene (**2l**) S24
2,5-Bis(1*H*-indol-3-yl)thiophene (**2p**) S26

^{1}H- and ^{13}C NMR spectra

2,5-diphenylthiophene (2a)

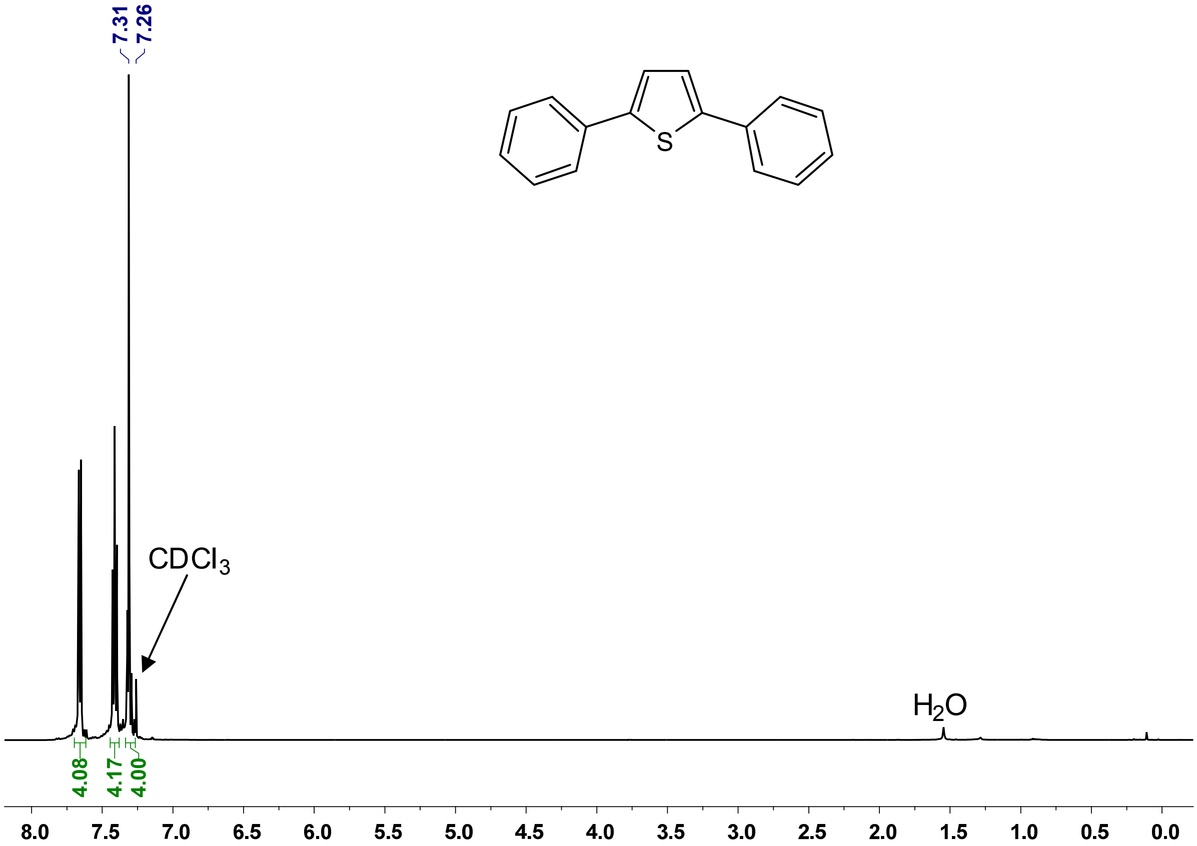

^{1}H NMR spectrum of **2a** recorded in $CDCl_3$ at 298 K (δ in ppm).

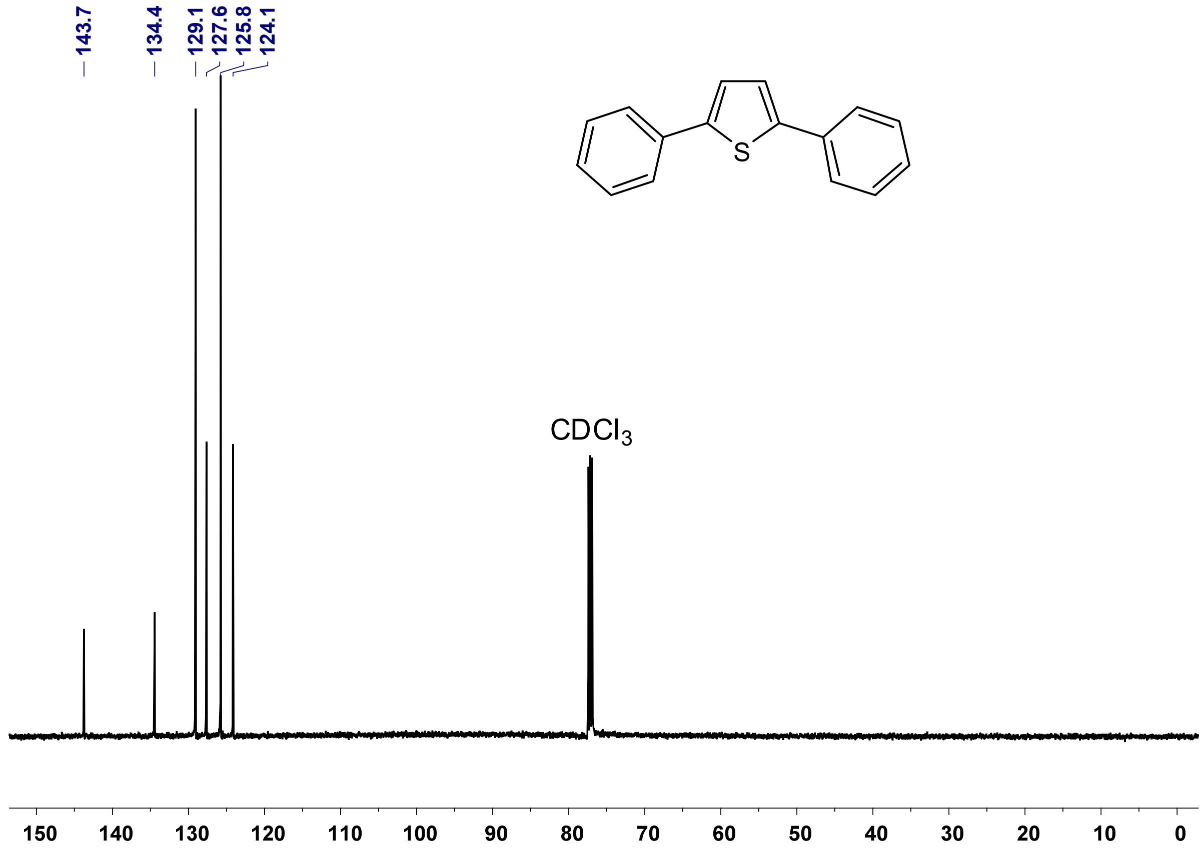

^{13}C NMR spectrum of **2a** recorded in $CDCl_3$ at 298 K (δ in ppm).

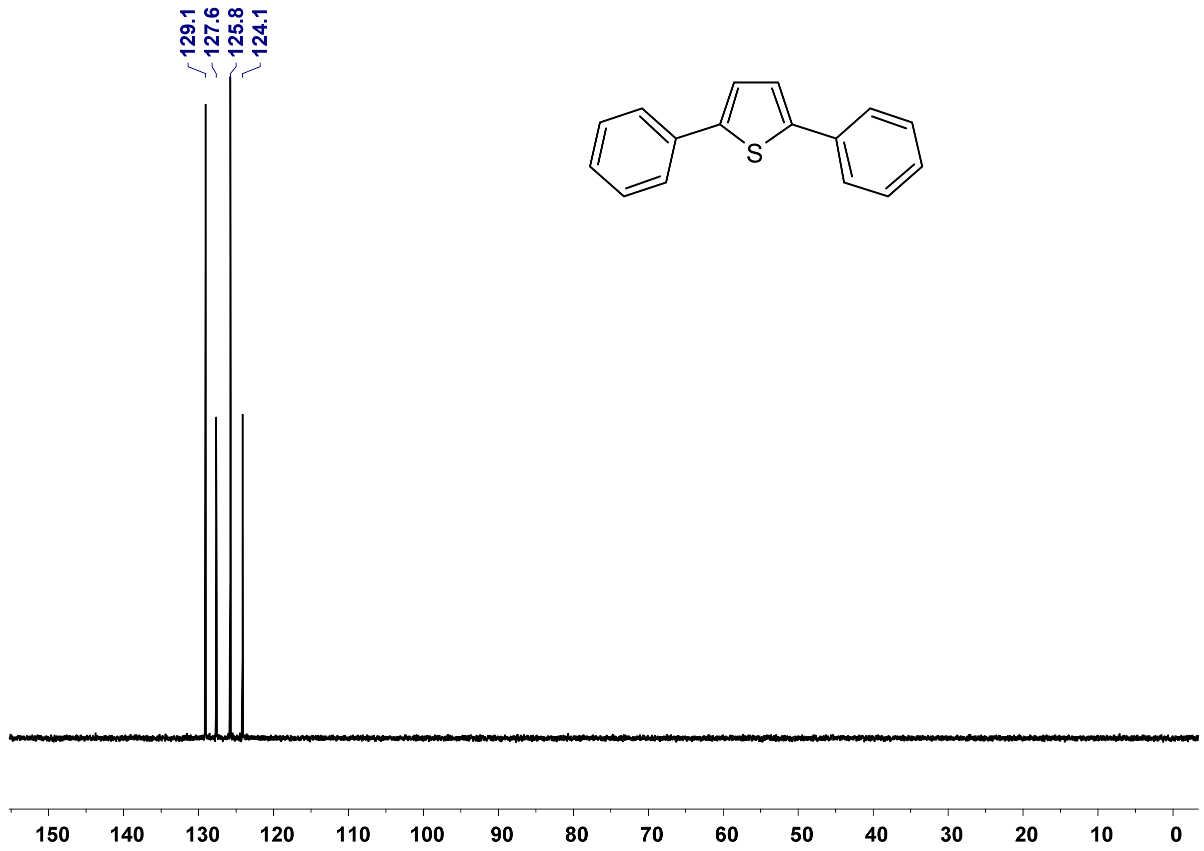

^{13}C-DEPT-135 NMR spectrum of **2a** recorded in $CDCl_3$ at 298 K (δ in ppm).

2,5-di(*o*-tolyl)thiophene (2b)

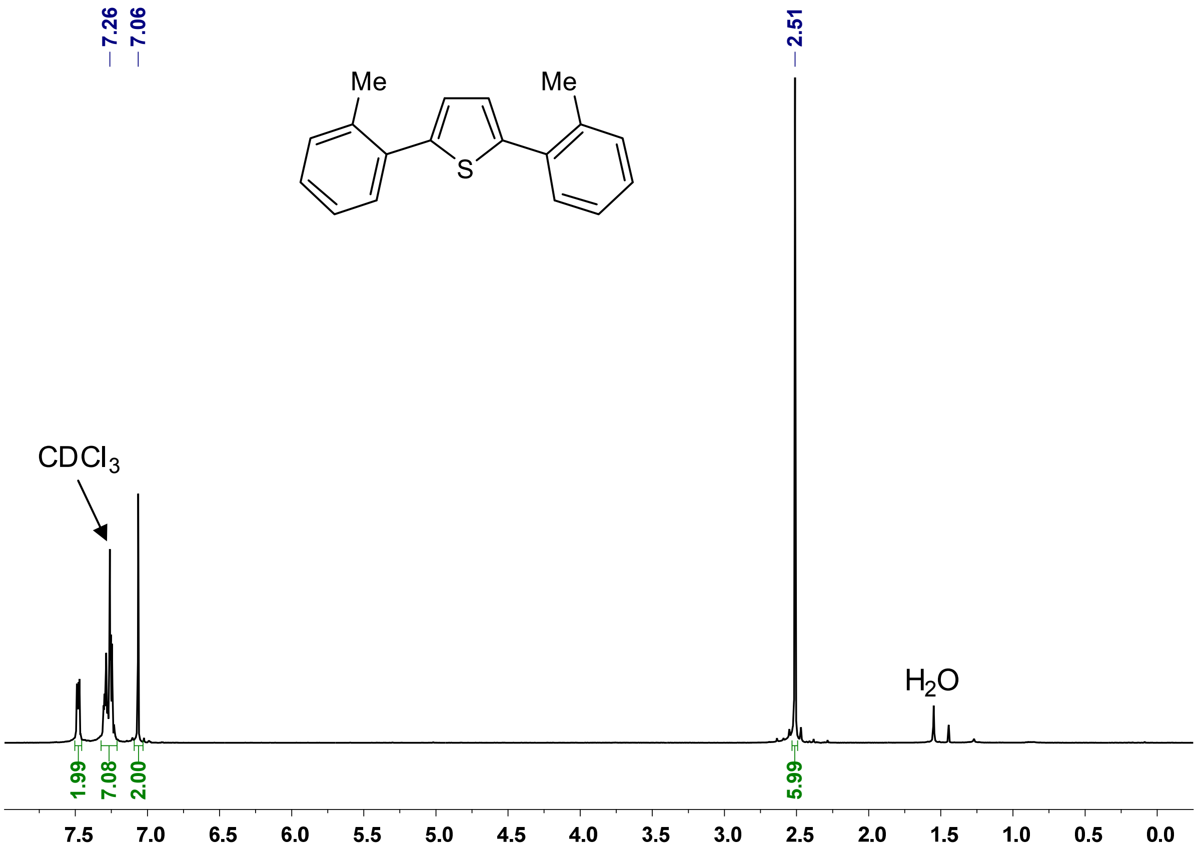

^{1}H NMR spectrum of **2b** recorded in $CDCl_3$ at 298 K (δ in ppm).

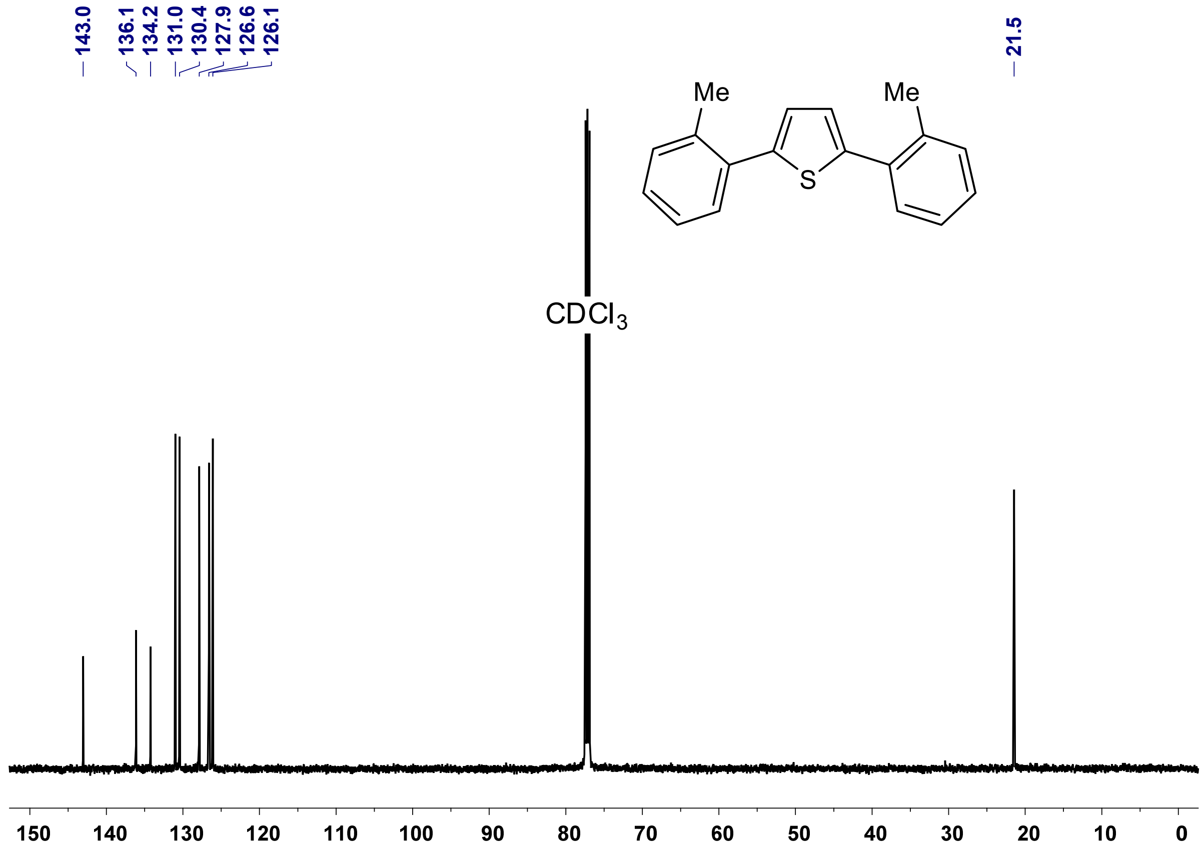

^{13}C NMR spectrum of **2b** recorded in $CDCl_3$ at 298 K (δ in ppm).

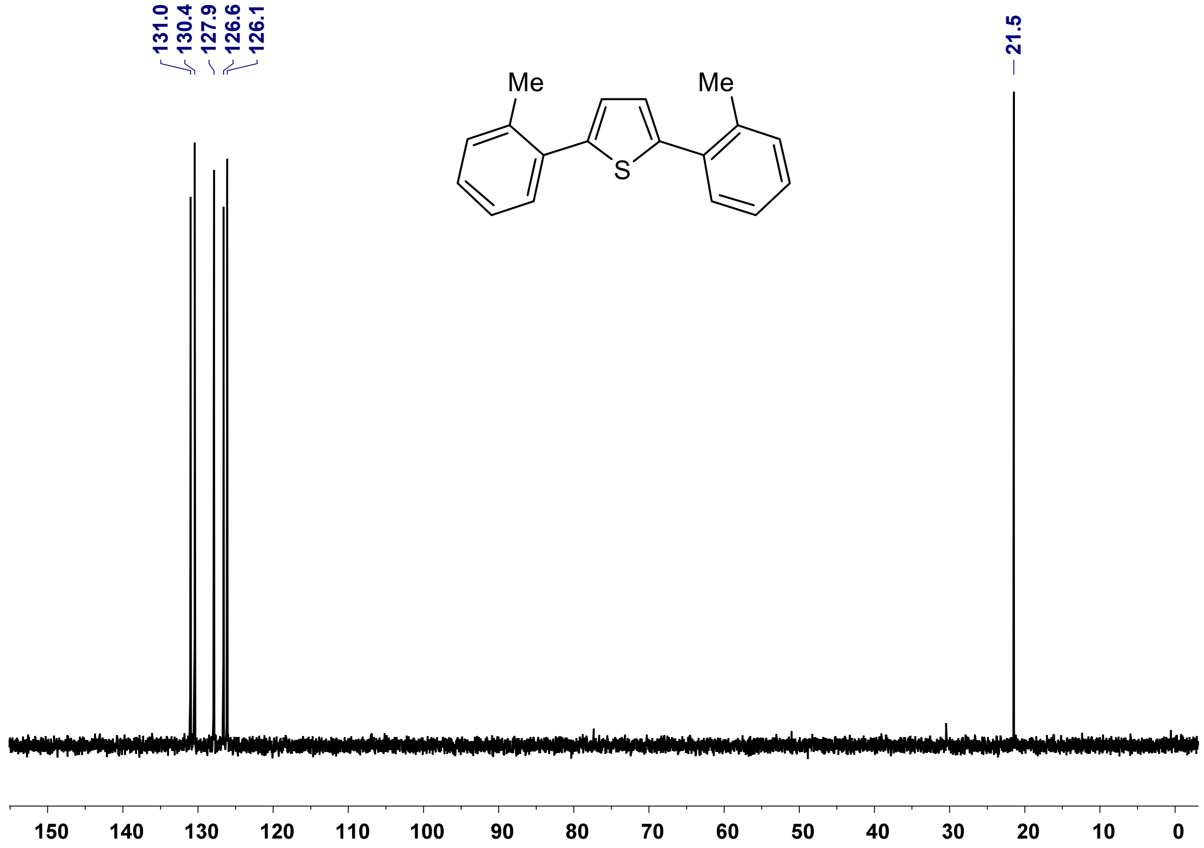

^{13}C DEPT-135 NMR spectrum of **2b** recorded in $CDCl_3$ at 298 K (δ in ppm).

2,5-bis(3-methoxyphenyl)thiophene (2c)

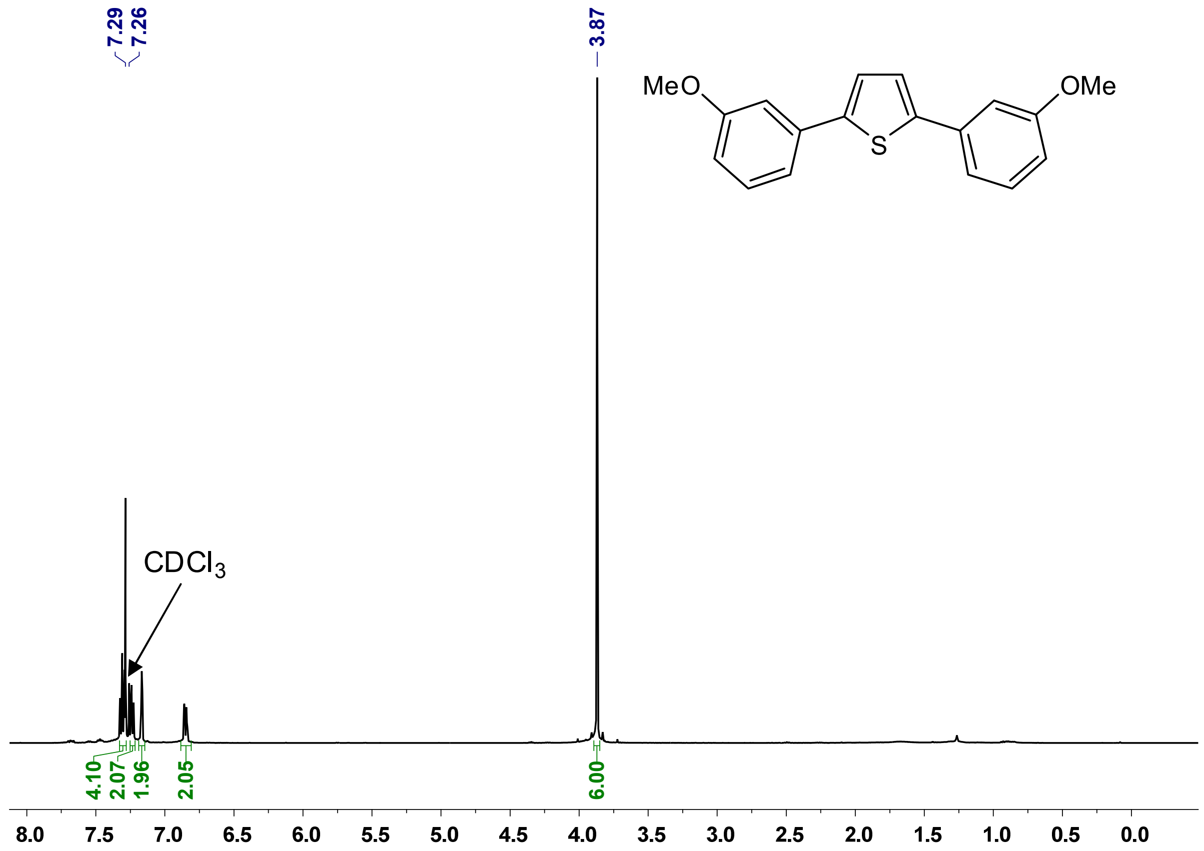

^{1}H NMR spectrum of **2c** recorded in $CDCl_3$ at 298 K (δ in ppm).

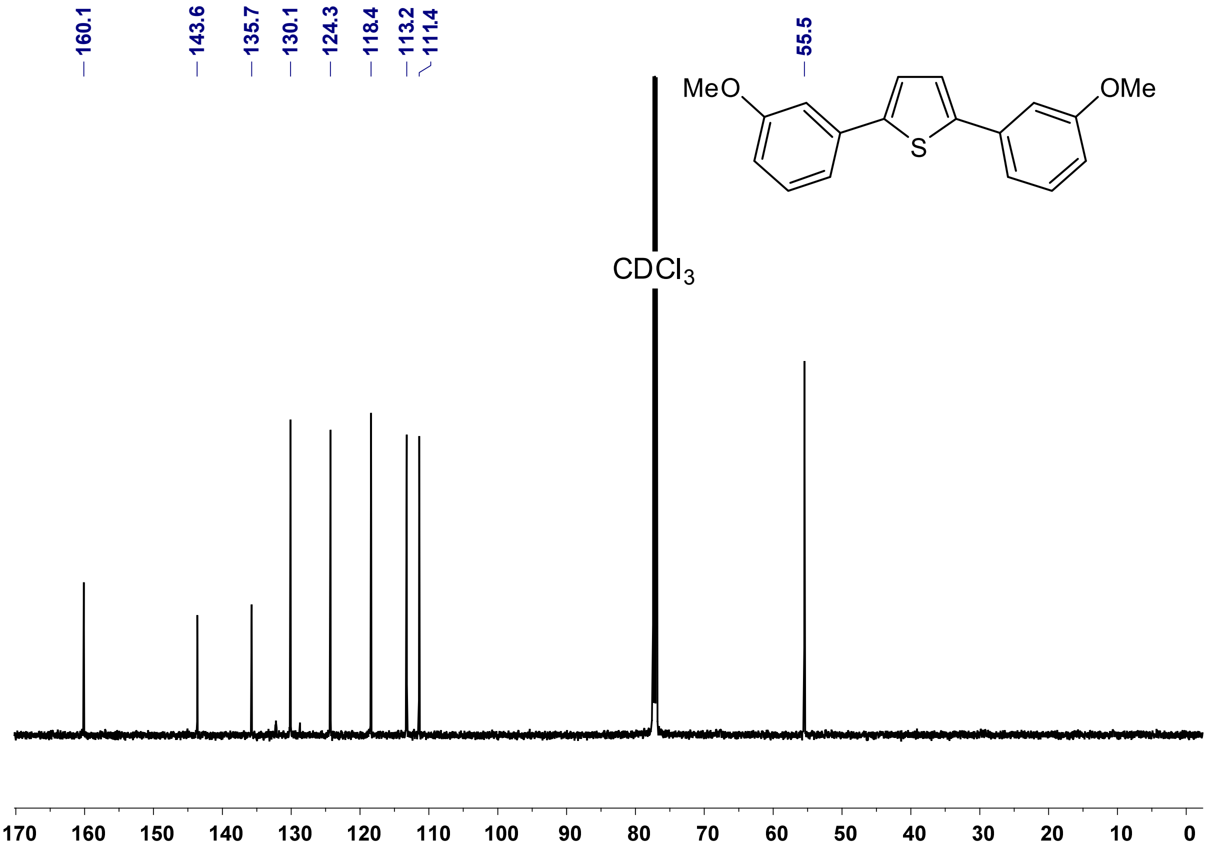

^{13}C NMR spectrum of **2e** recorded in $CDCl_3$ at 298 K (δ in ppm).

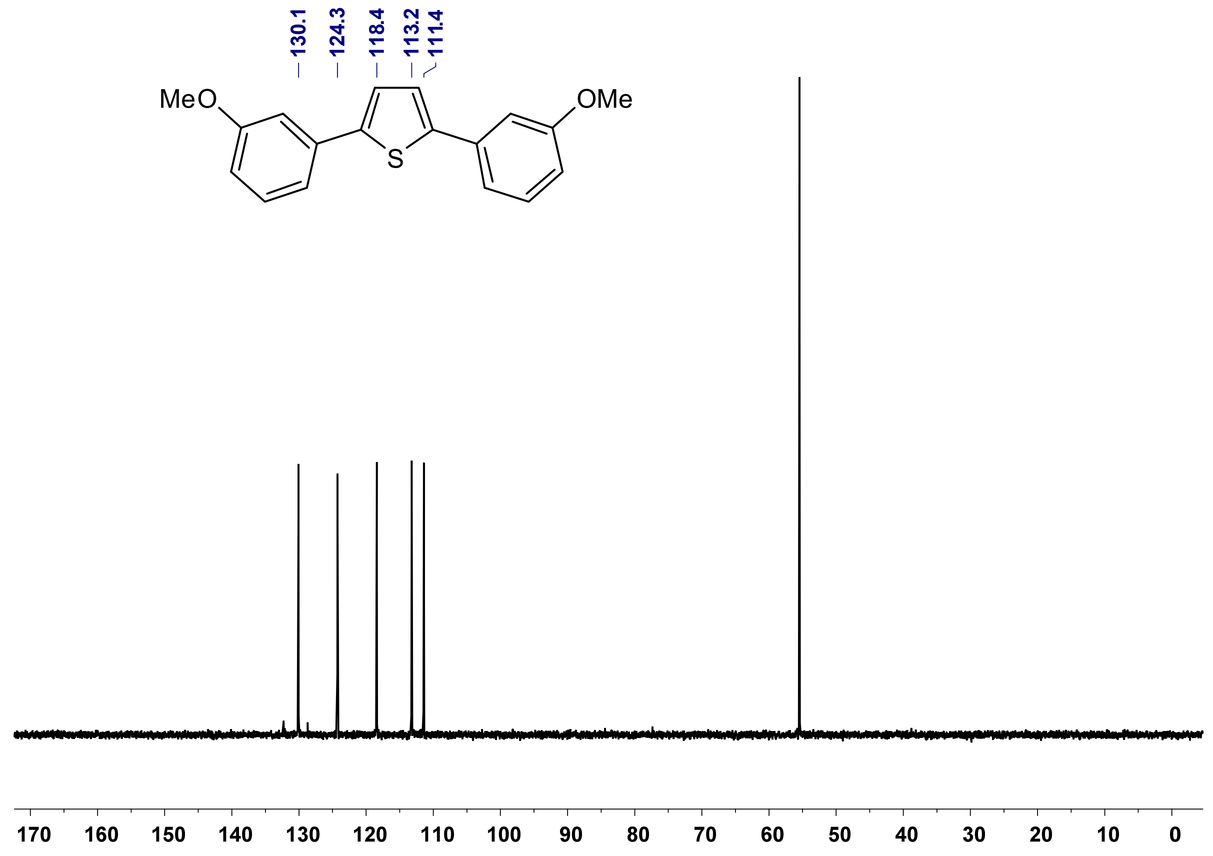

^{13}C DEPT-135 NMR spectrum of **2i** recorded in $CDCl_3$ at 298 K (δ in ppm).

2,5-bis(3-bromophenyl)thiophene (2d)

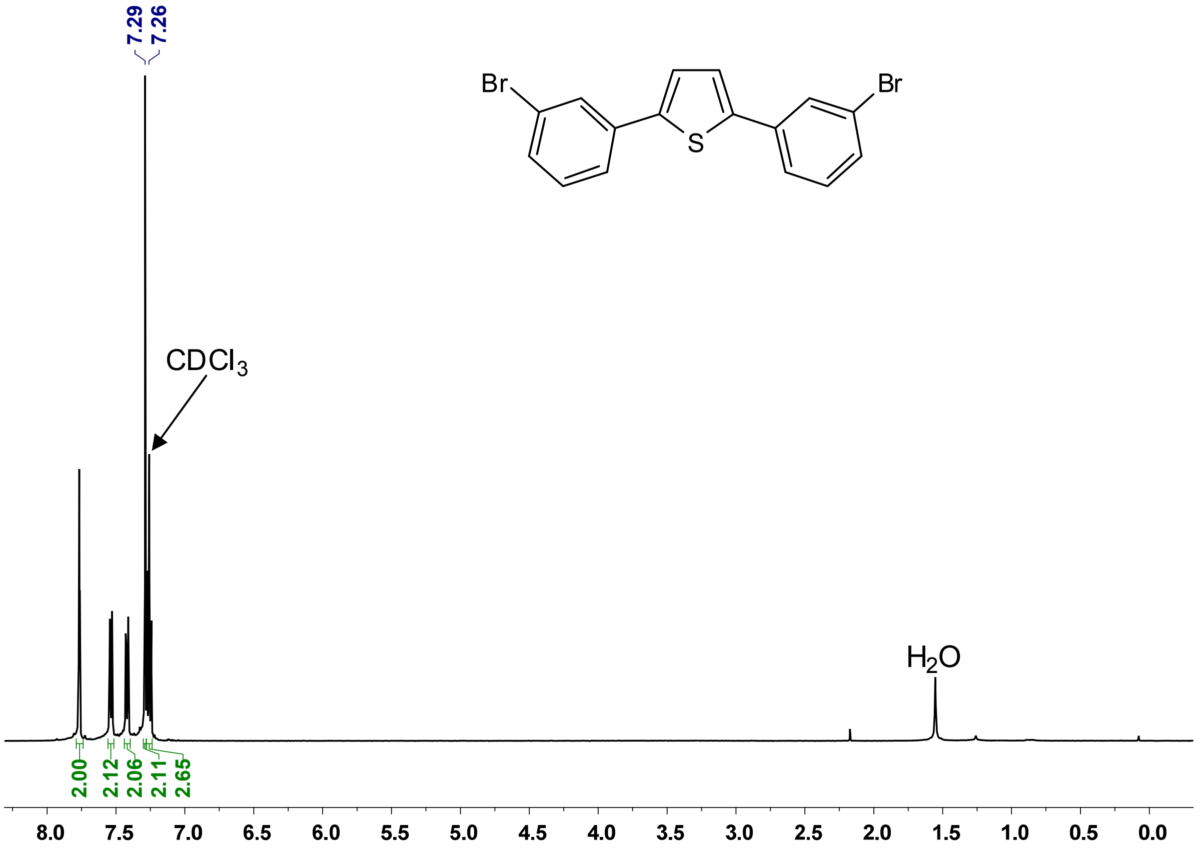

^{1}H NMR spectrum of **2d** recorded in $CDCl_3$ at 298 K (δ in ppm).

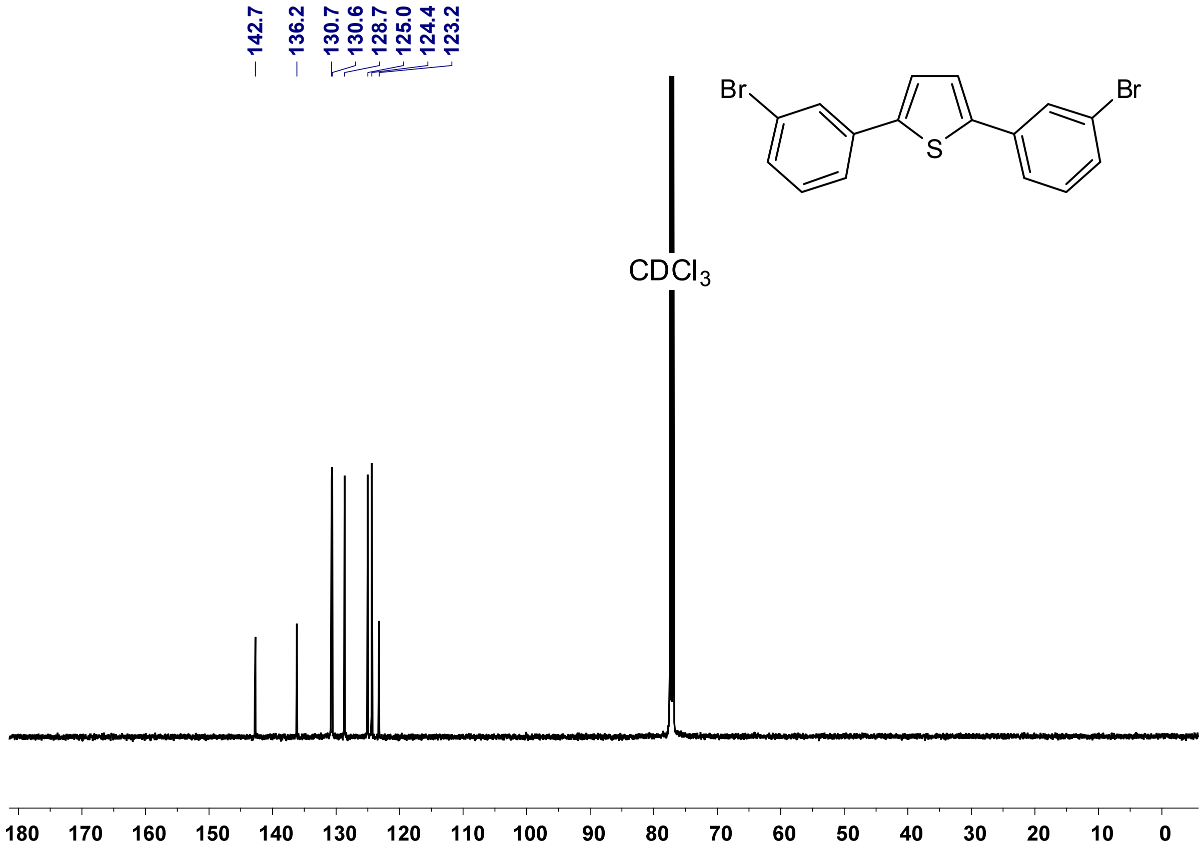

^{13}C NMR spectrum of **2d** recorded in $CDCl_3$ at 298 K (δ in ppm).

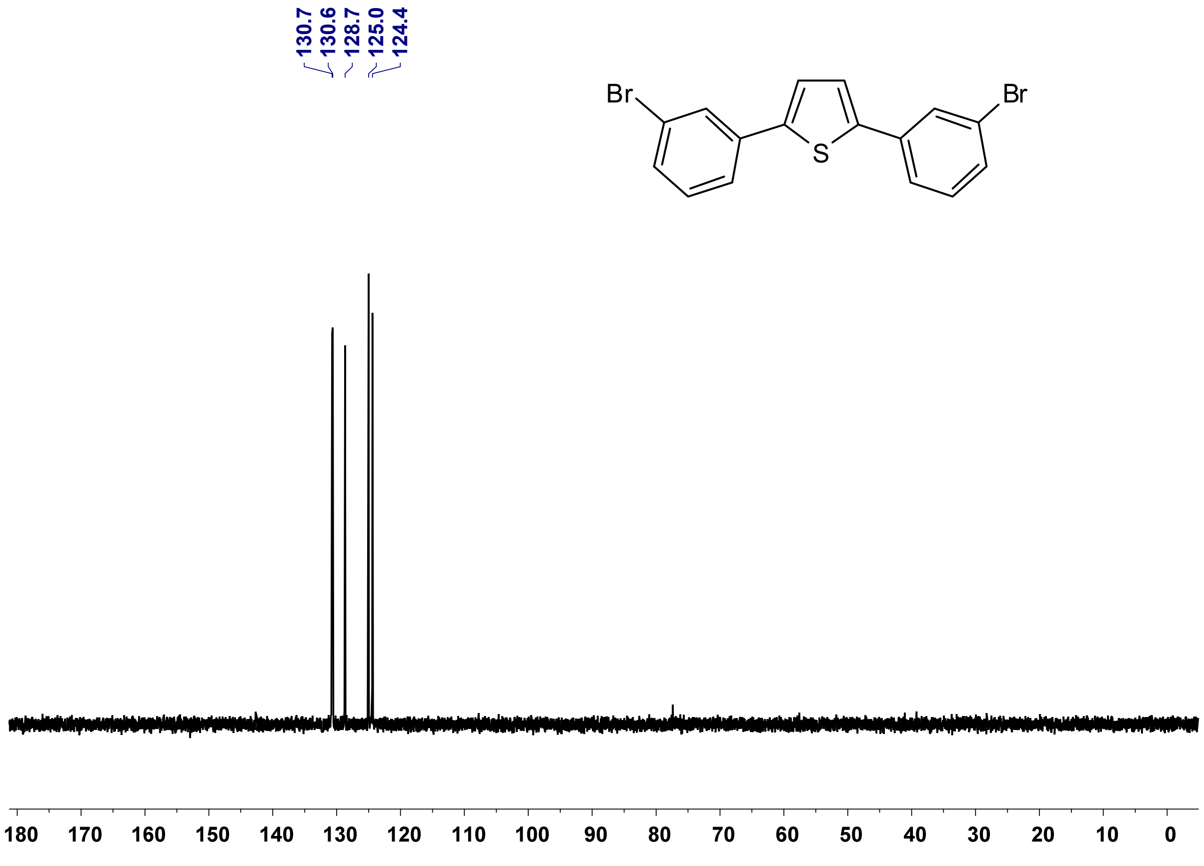

^{13}C DEPT-135 NMR spectrum of **2i** recorded in $CDCl_3$ at 298 K (δ in ppm).

2,5-bis(3-chlorophenyl)thiophene (2e)

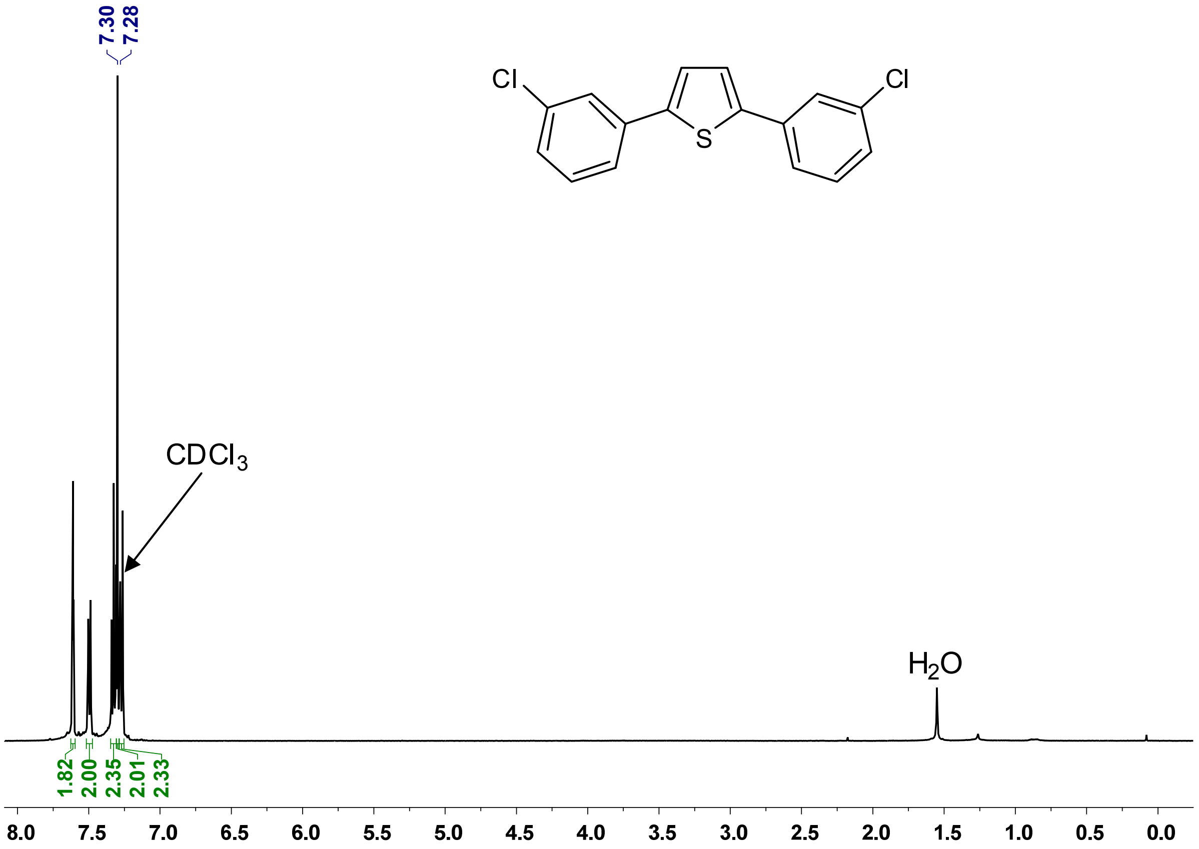

^{1}H NMR spectrum of **2e** recorded in $CDCl_3$ at 299 K (δ in ppm).

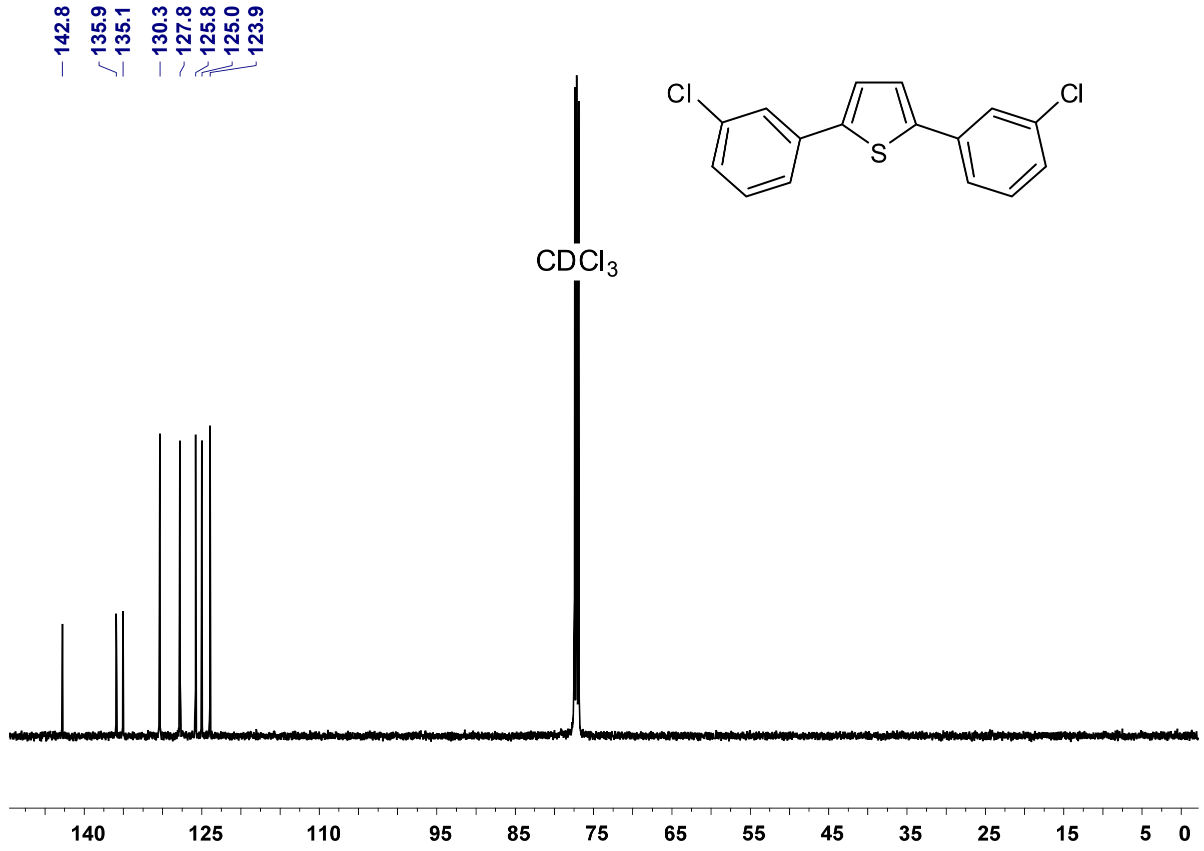

^{13}C NMR spectrum of **2e** recorded in $CDCl_3$ at 299 K (δ in ppm).

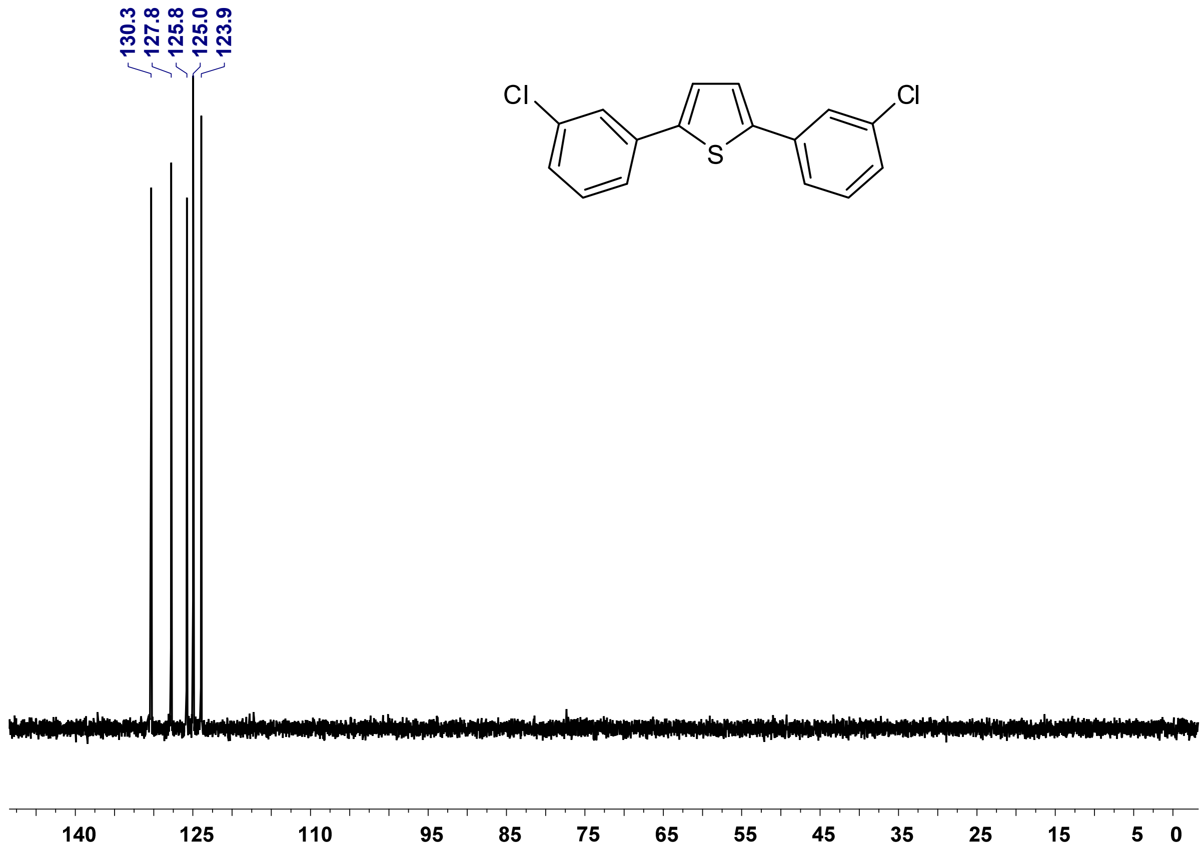

^{13}C DEPT-135 NMR spectrum of **2e** recorded in $CDCl_3$ at 299 K (δ in ppm).

3,3'-(thiophene-2,5-diyl)diphenol (2f)

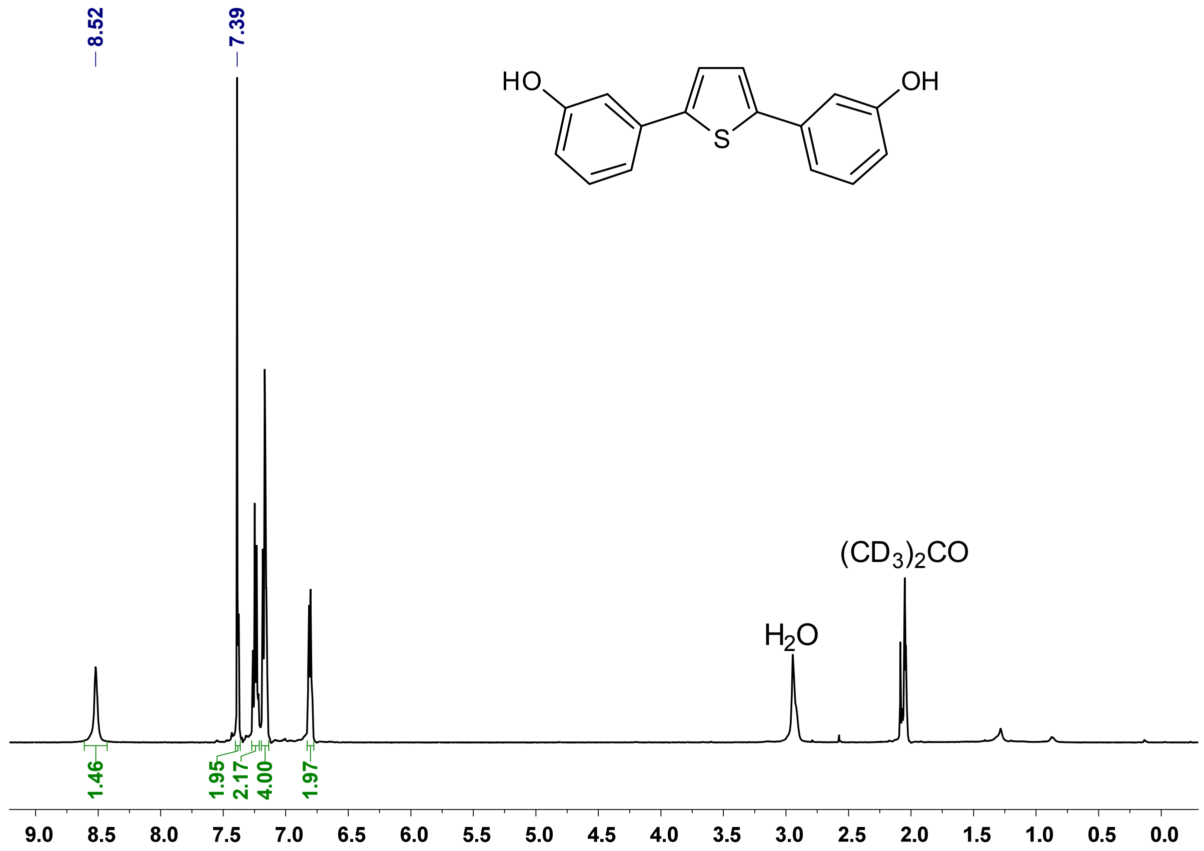

^{1}H NMR spectrum of **2f** recorded in acetone-d_6 at 298 K (δ in ppm).

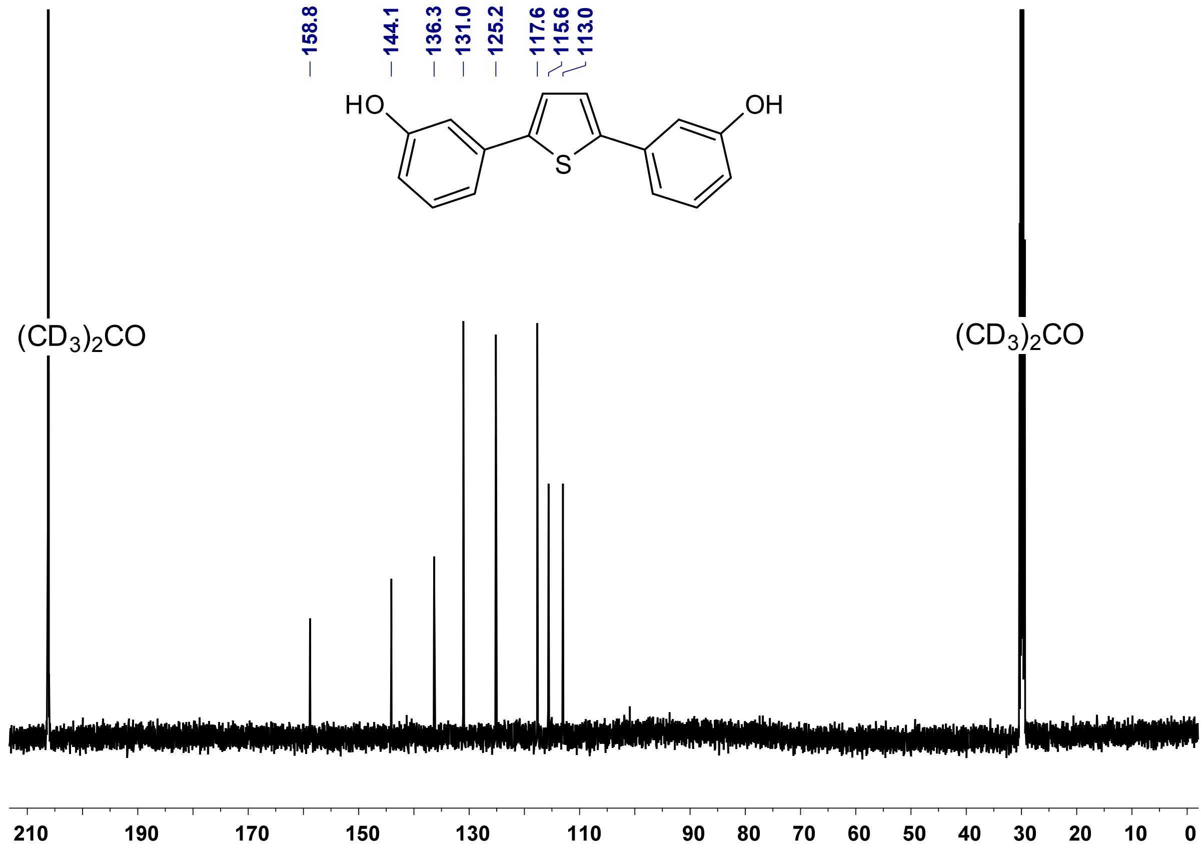

^{13}C NMR spectrum of **2f** recorded in acetone-d$_6$ at 298 K (δ in ppm).

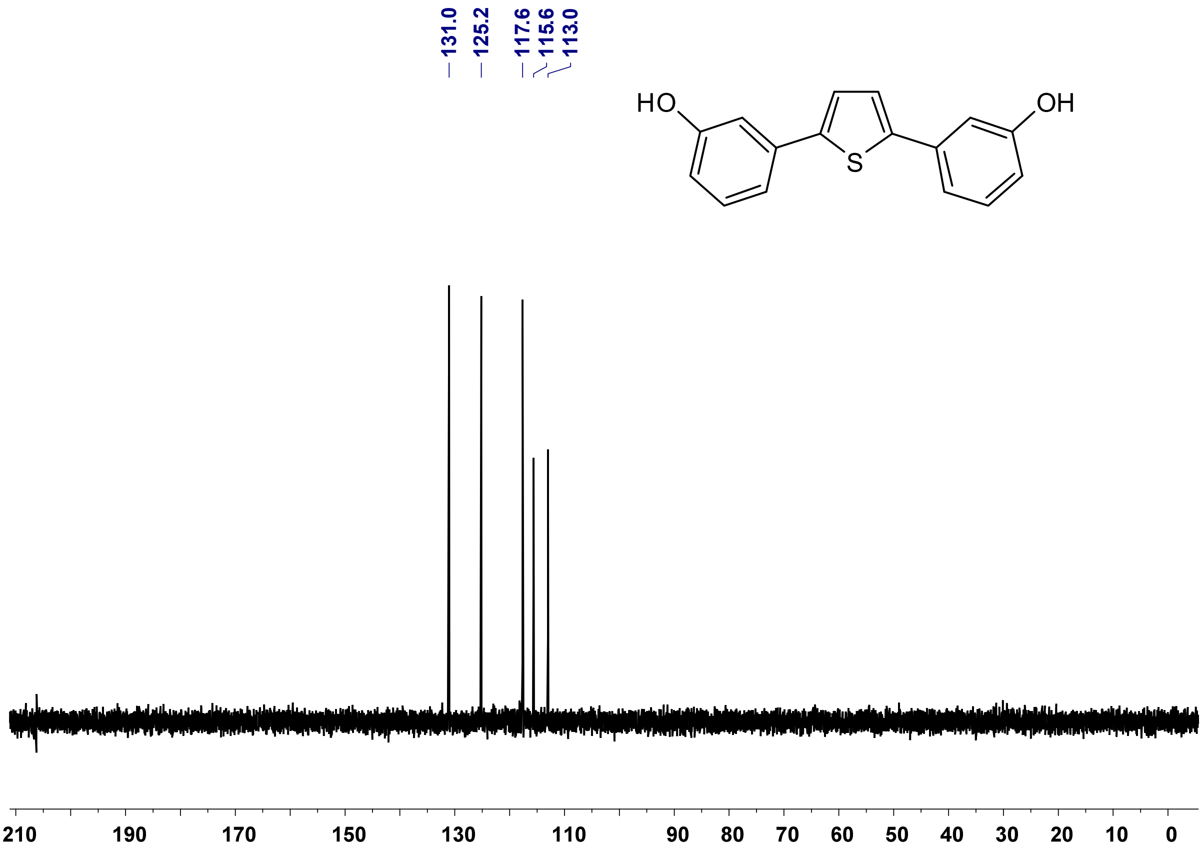

^{13}C DEPT-135 NMR spectrum of **2f** recorded in acetone-d$_6$ at 298 K (δ in ppm).

3,3'-(thiophene-2,5-diyl)dianiline (2g)

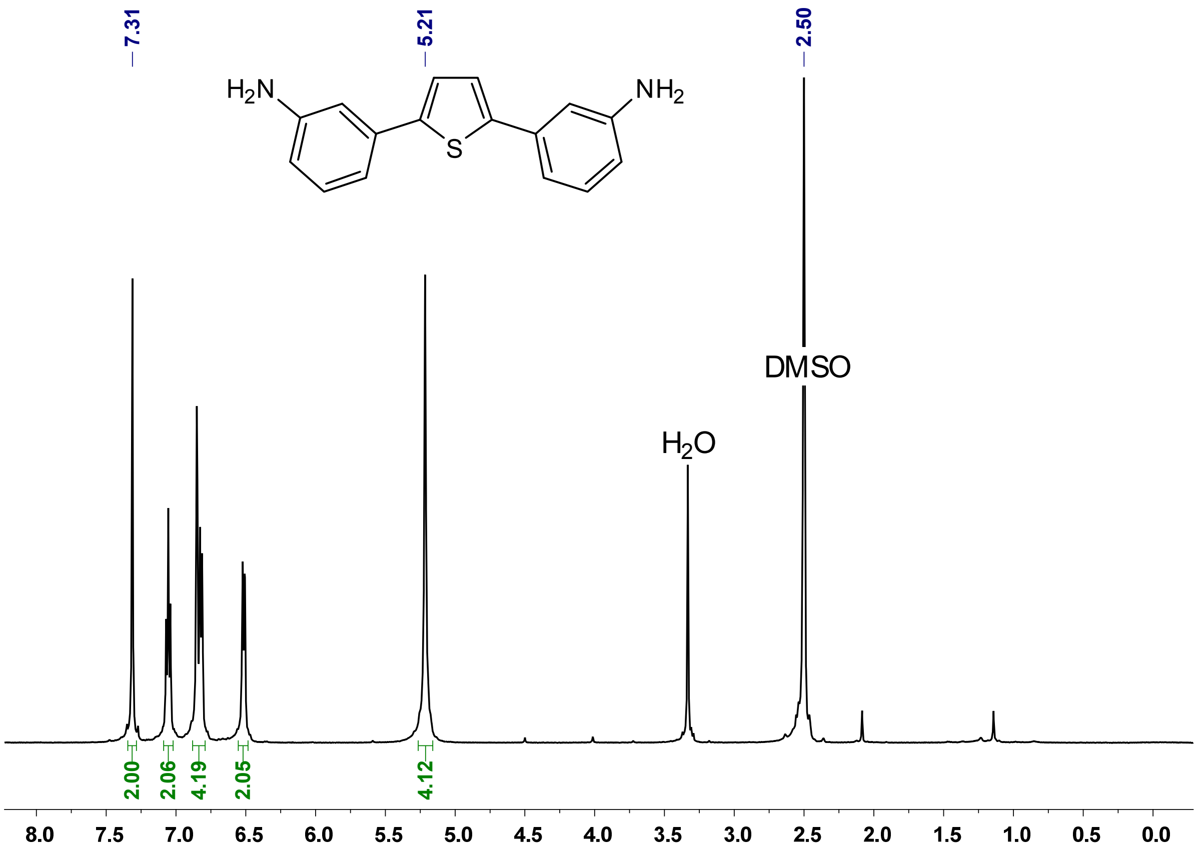

^1H NMR spectrum of **2g** recorded in DMSO-d_6 at 298 K (δ in ppm).

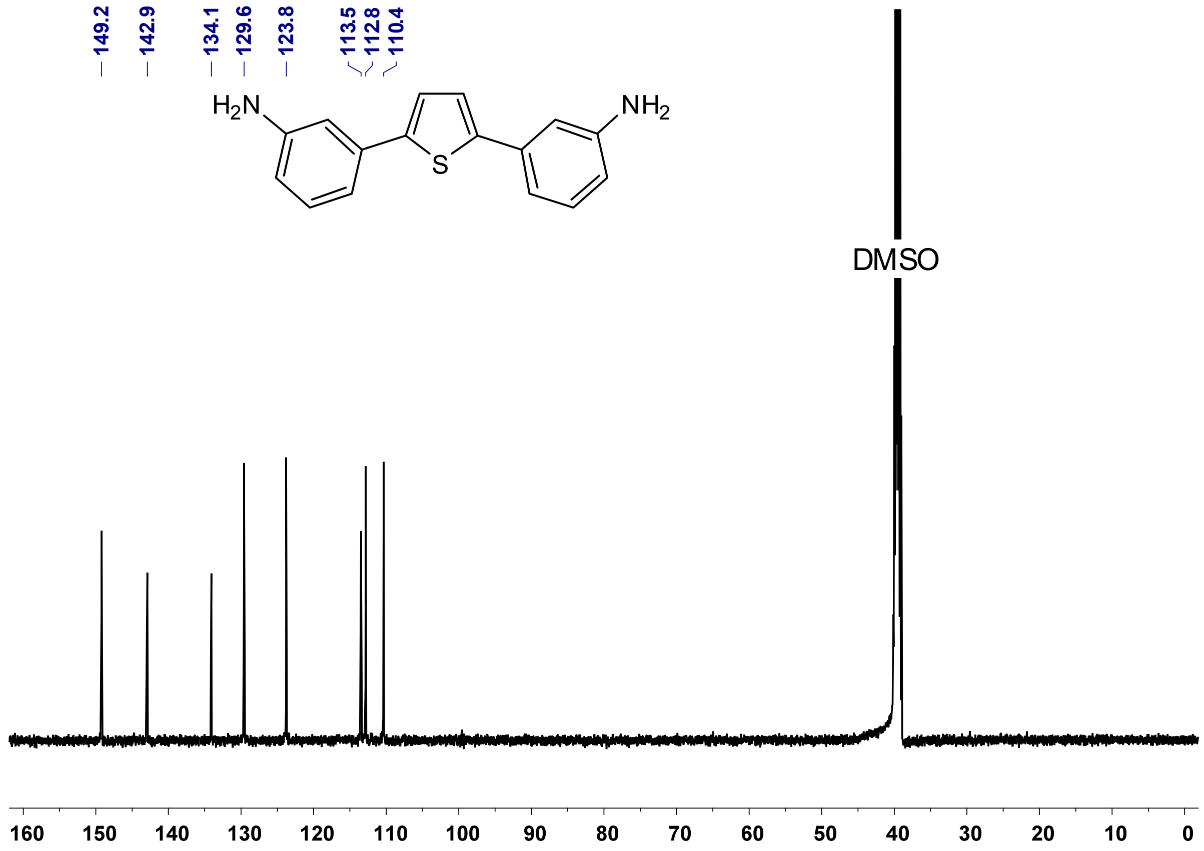

^{13}C NMR spectrum of **2g** recorded in DMSO-d_6 at 298 K (δ in ppm).

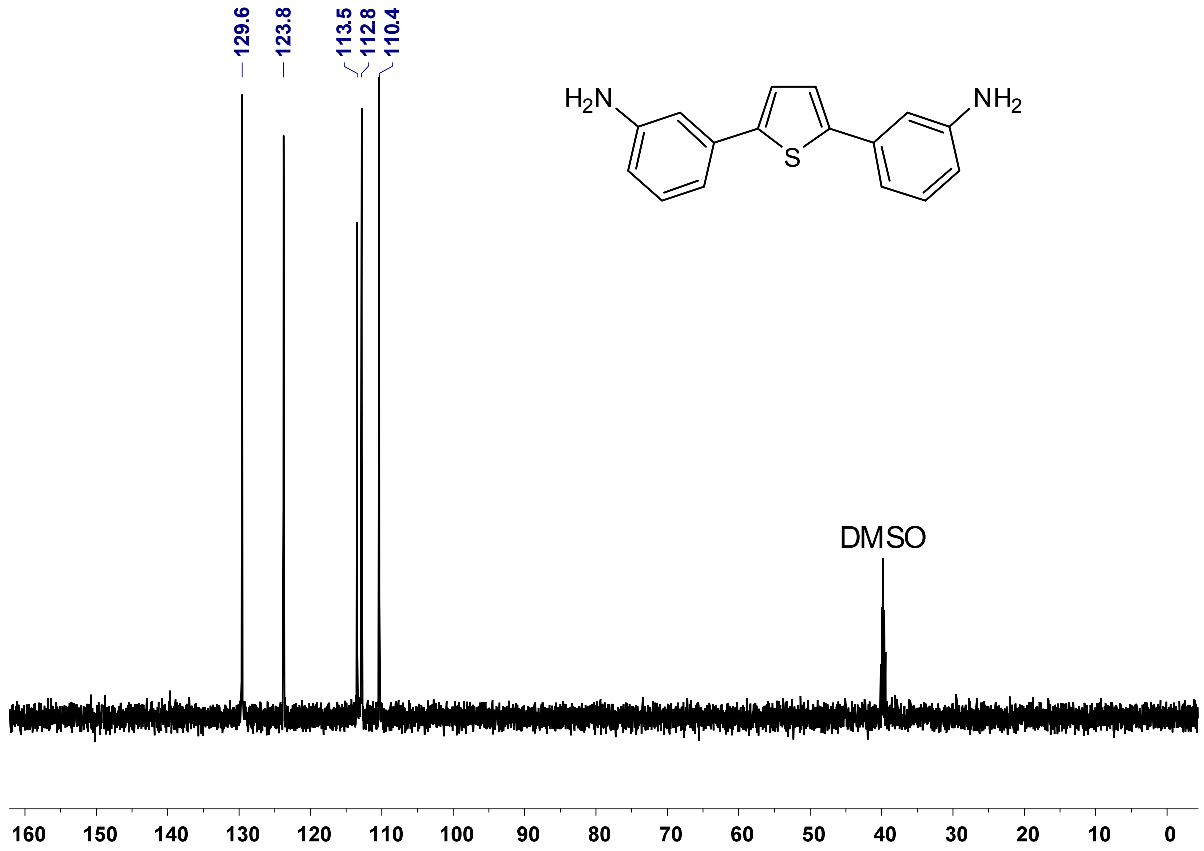

^{13}C DEPT-135 NMR spectrum of **2g** recorded in DMSO-d_6 at 298 K (δ in ppm).

2,5-bis(4-chlorophenyl)thiophene (2h)

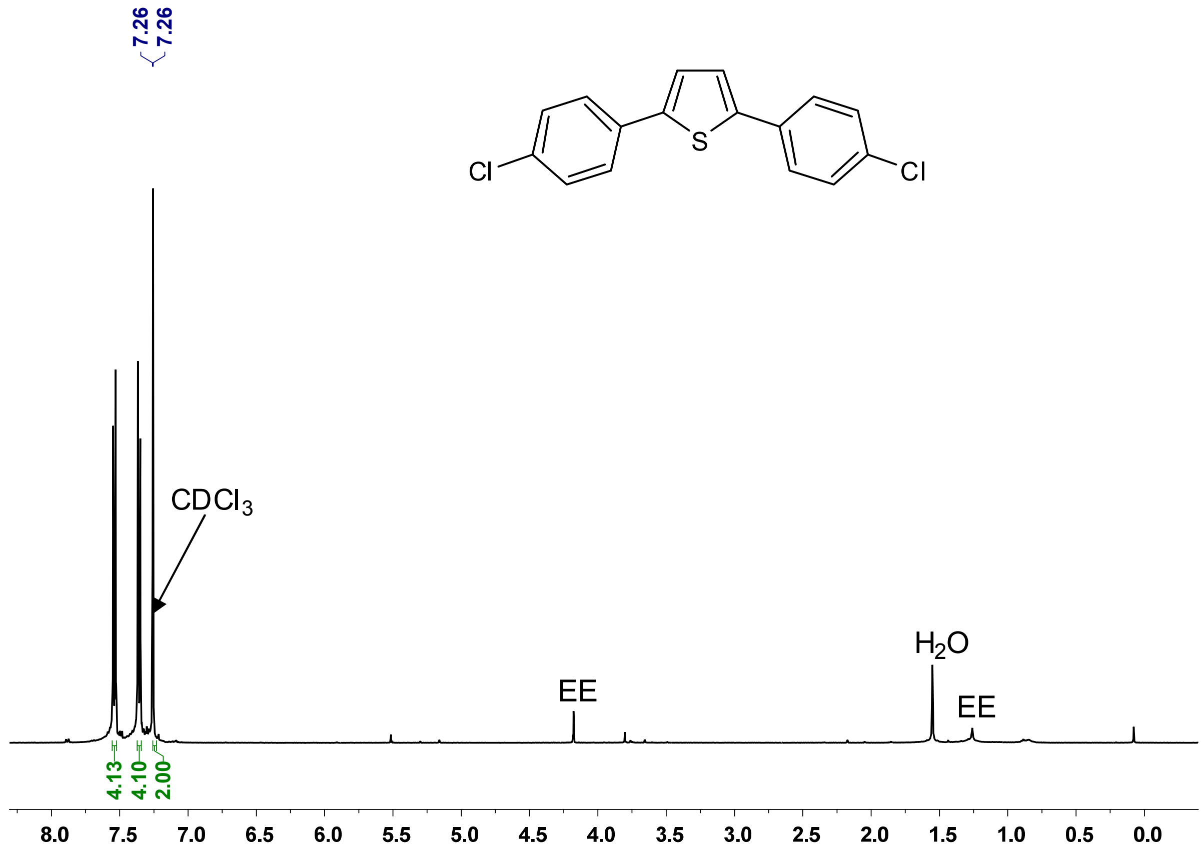

^{1}H NMR spectrum of **2h** recorded in $CDCl_3$ at 298 K (δ in ppm).

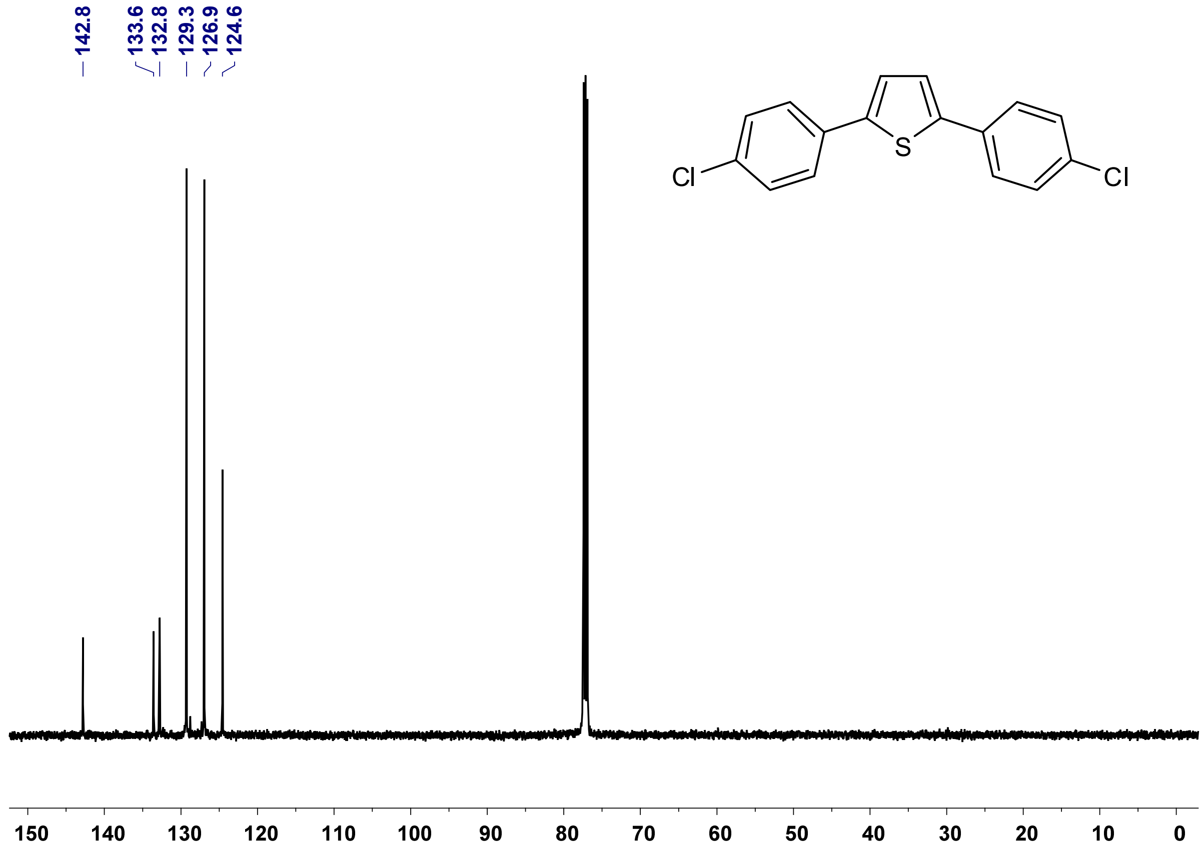

^{13}C NMR spectrum of **2h** recorded in $CDCl_3$ at 298 K (δ in ppm).

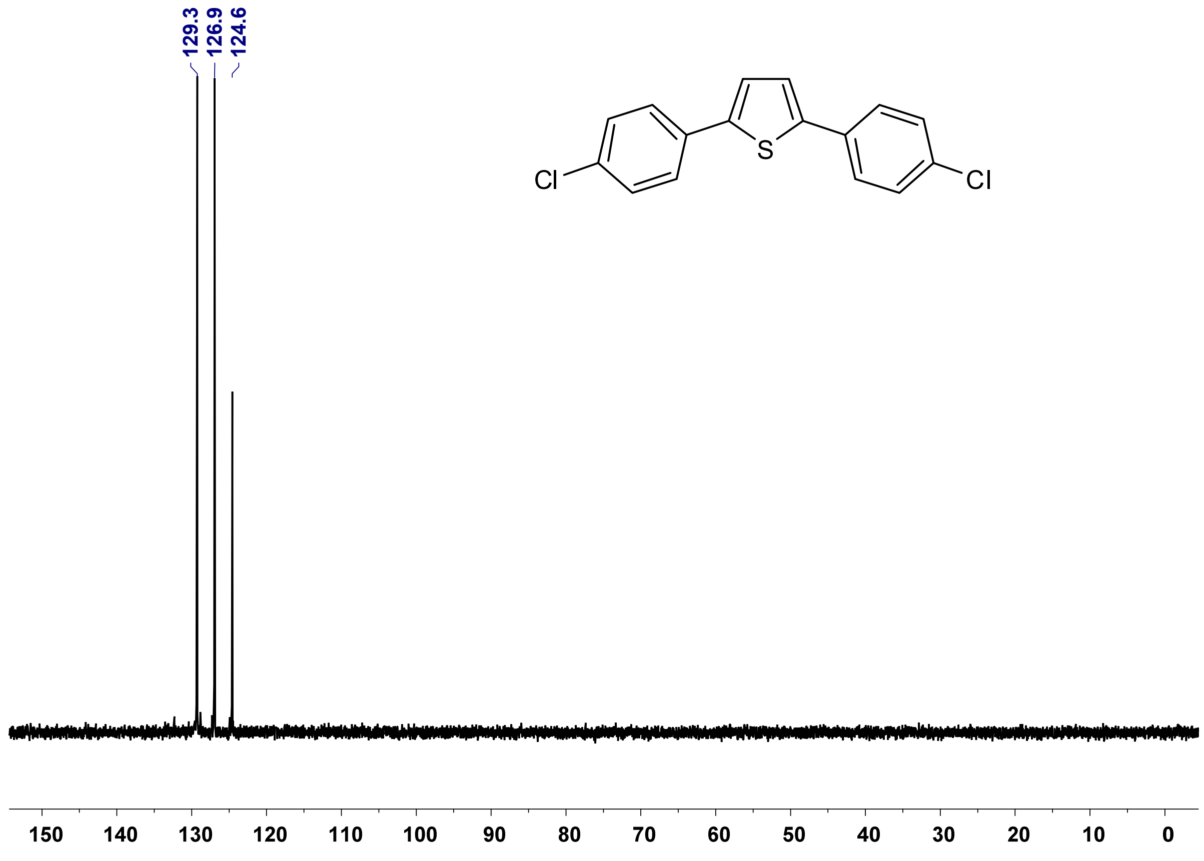

^{13}C DEPT-135 NMR spectrum of **2h** recorded in $CDCl_3$ at 298 K (δ in ppm).

2,5-bis(4-fluorophenyl)thiophene (2i)

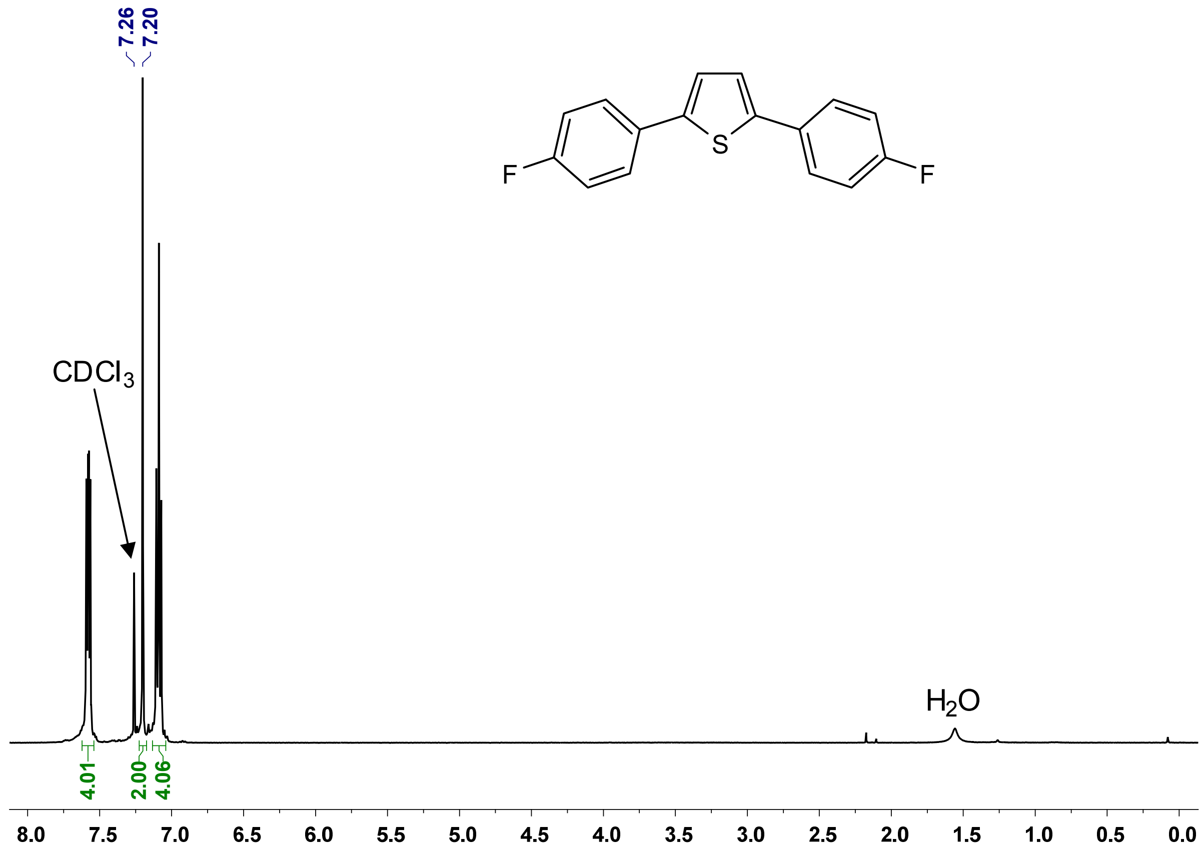

^{1}H NMR spectrum of **2i** recorded in $CDCl_3$ at 298 K (δ in ppm).

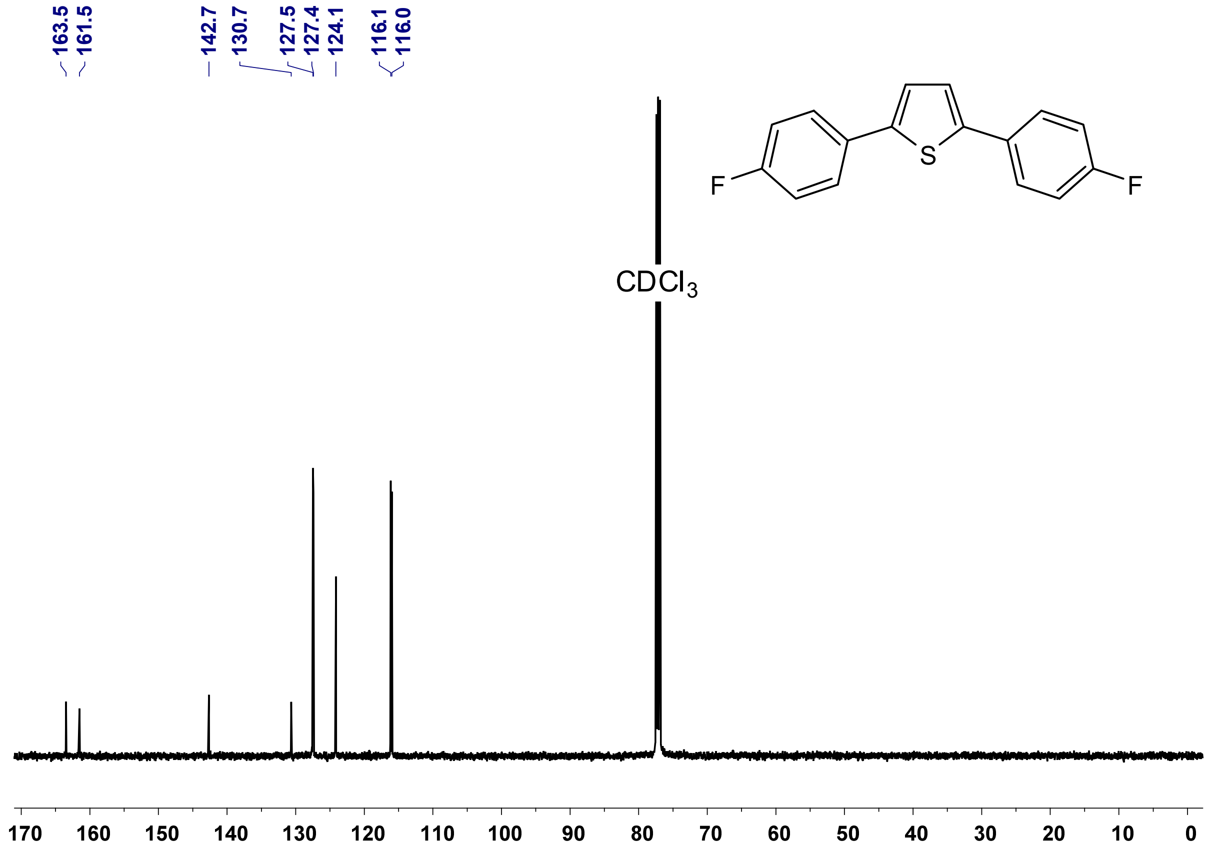

^{13}C NMR spectrum of **2i** recorded in $CDCl_3$ at 298 K (δ in ppm).

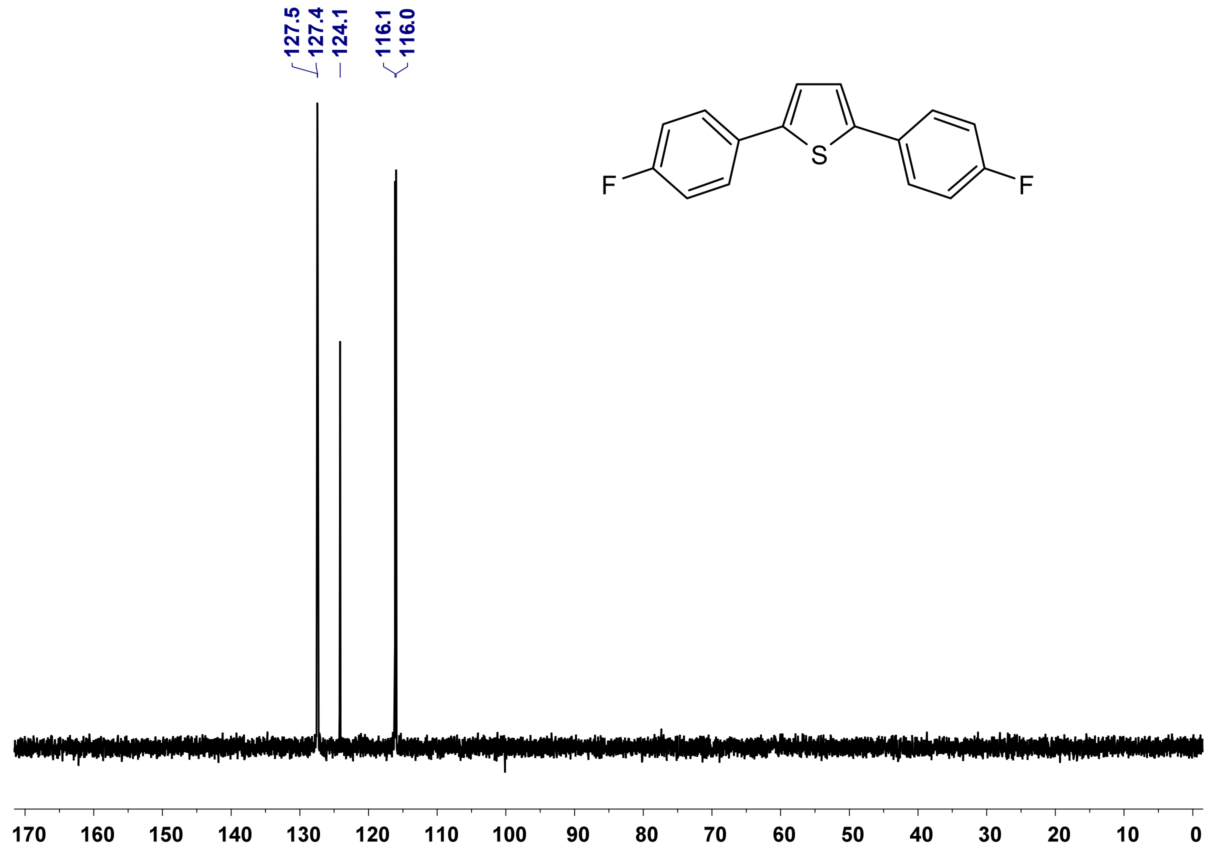

^{13}C DEPT-135 NMR spectrum of **2i** recorded in $CDCl_3$ at 298 K (δ in ppm).

2,5-di(pyridin-4-yl)thiophene (2j)

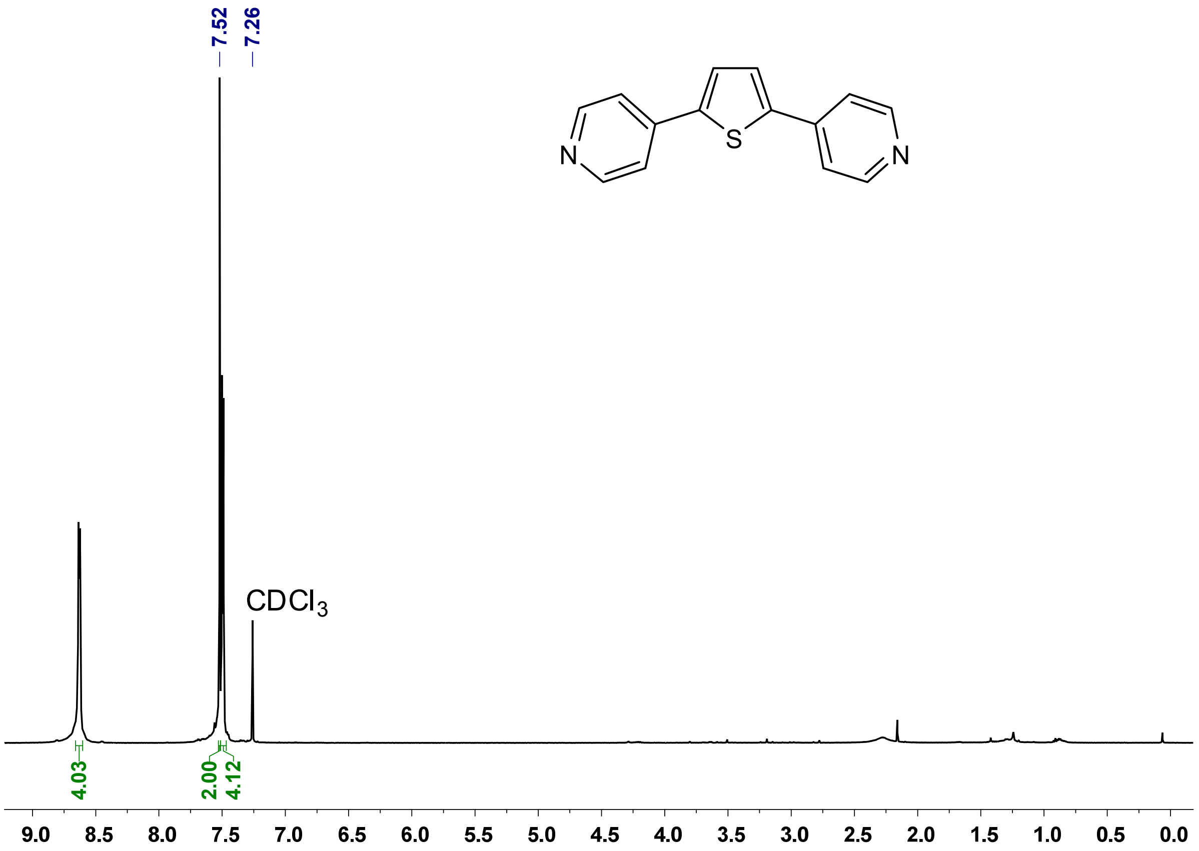

^{1}H NMR spectrum of **2j** recorded in $CDCl_3$ at 298 K (δ in ppm).

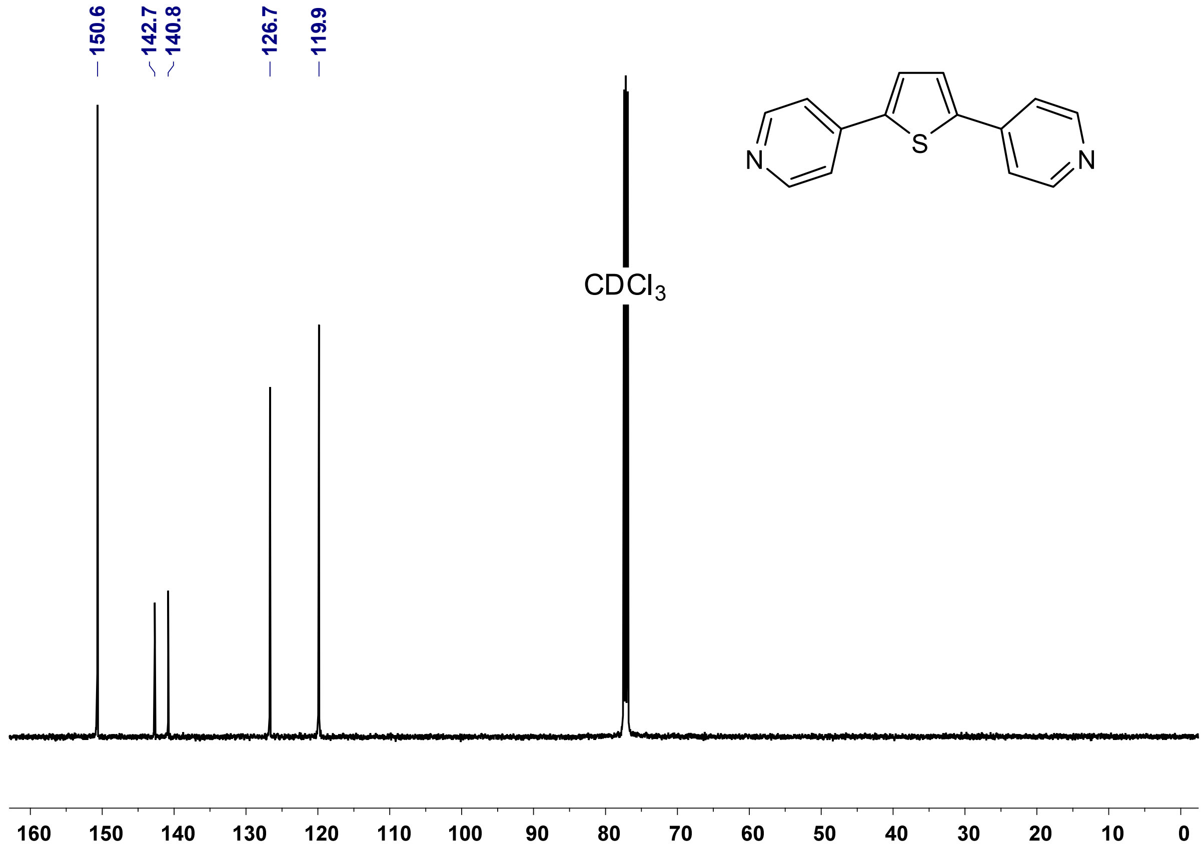

^{13}C NMR spectrum of **2j** recorded in $CDCl_3$ at 298 K (δ in ppm).

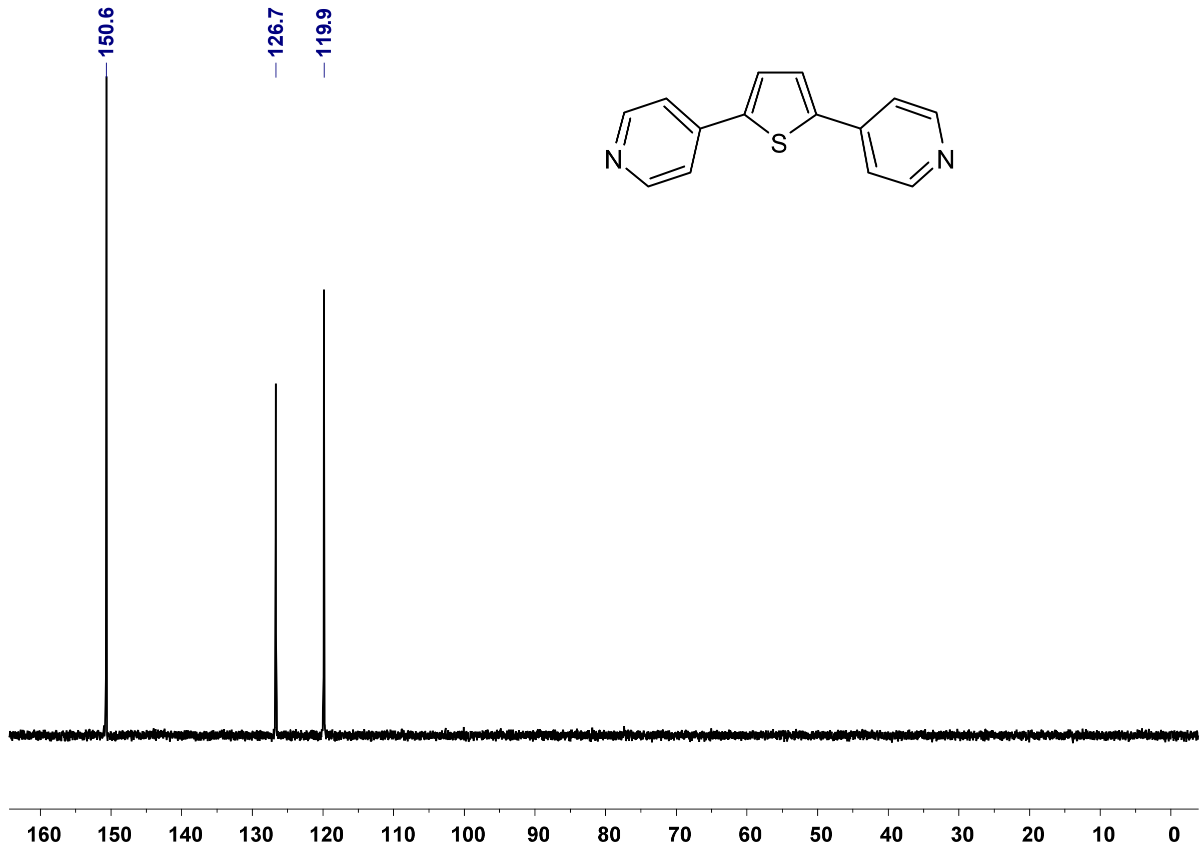

^{13}C DEPT-135 NMR spectrum of **2j** recorded in $CDCl_3$ at 298 K (δ in ppm).

[2,2':5',2'']-terthiophene (2k)

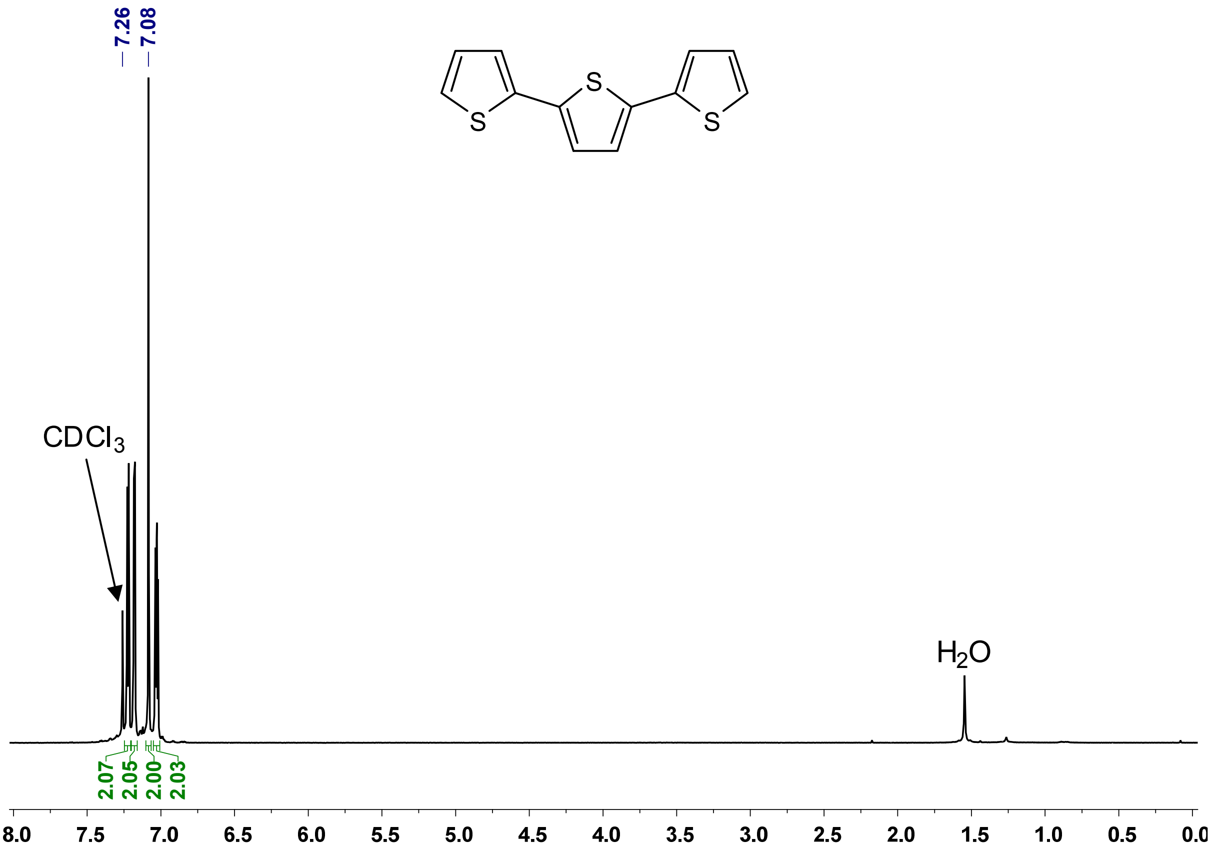

^{1}H NMR spectrum of **2k** recorded in $CDCl_3$ at 298 K (δ in ppm).

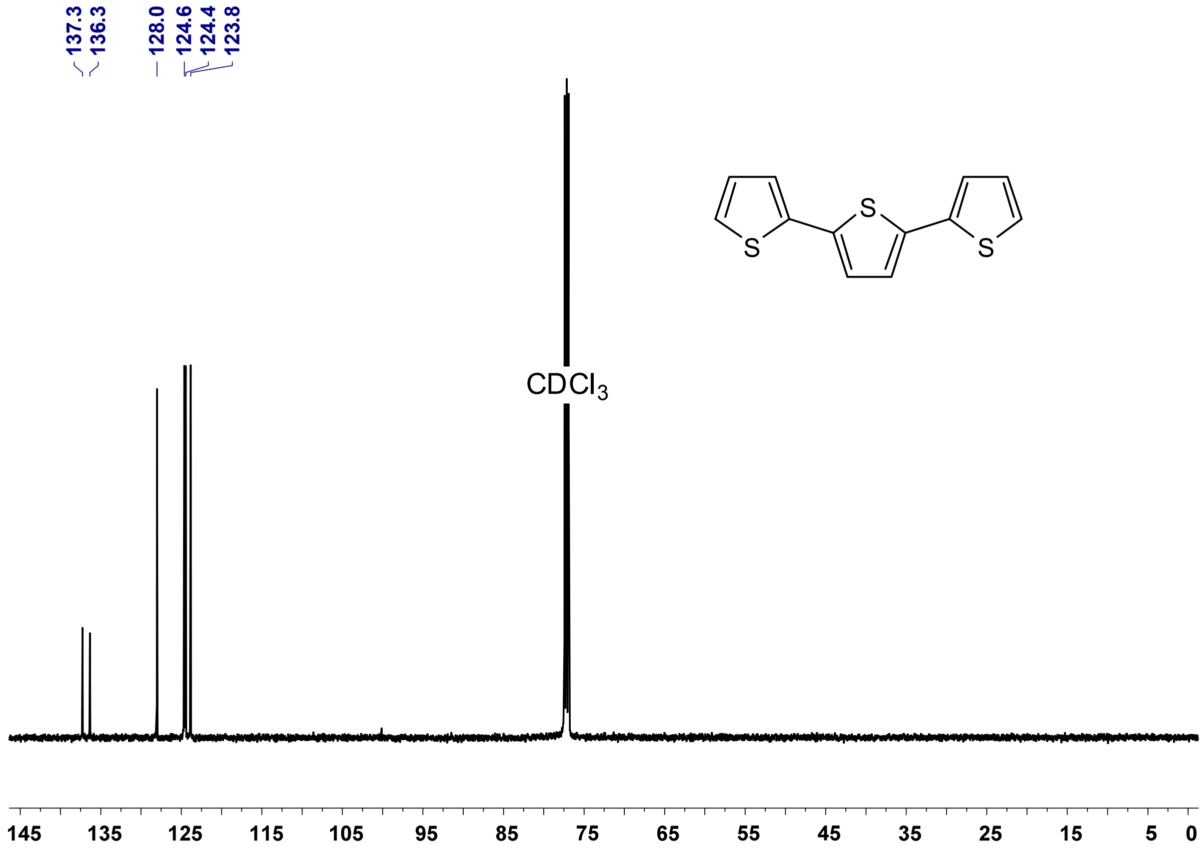

^{13}C NMR spectrum of **2k** recorded in $CDCl_3$ at 298 K (δ in ppm).

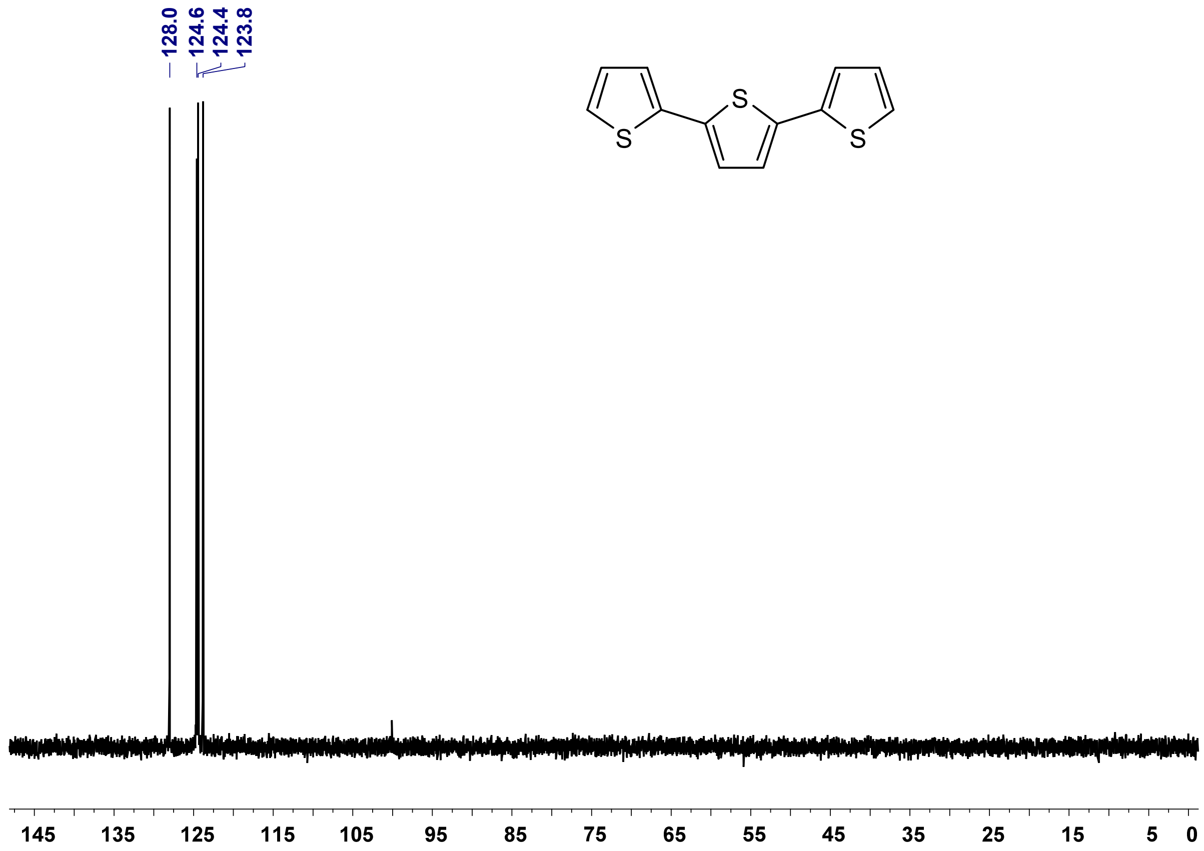

^{13}C DEPT-135 NMR spectrum of **2k** recorded in $CDCl_3$ at 298 K (δ in ppm).

2,5-di(naphthalen-1-yl)thiophene (2l)

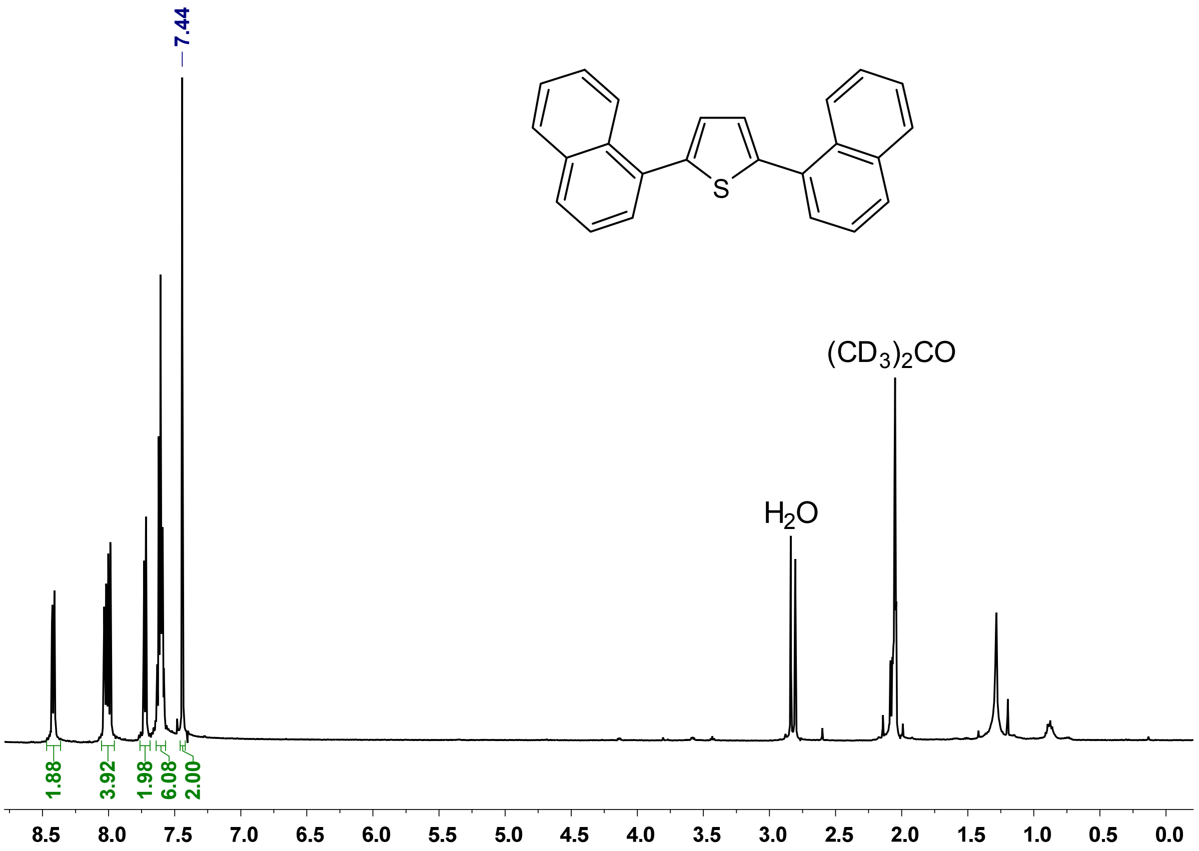

^{1}H NMR spectrum of **2l** recorded in acetone-d_6 at 298 K (δ in ppm).

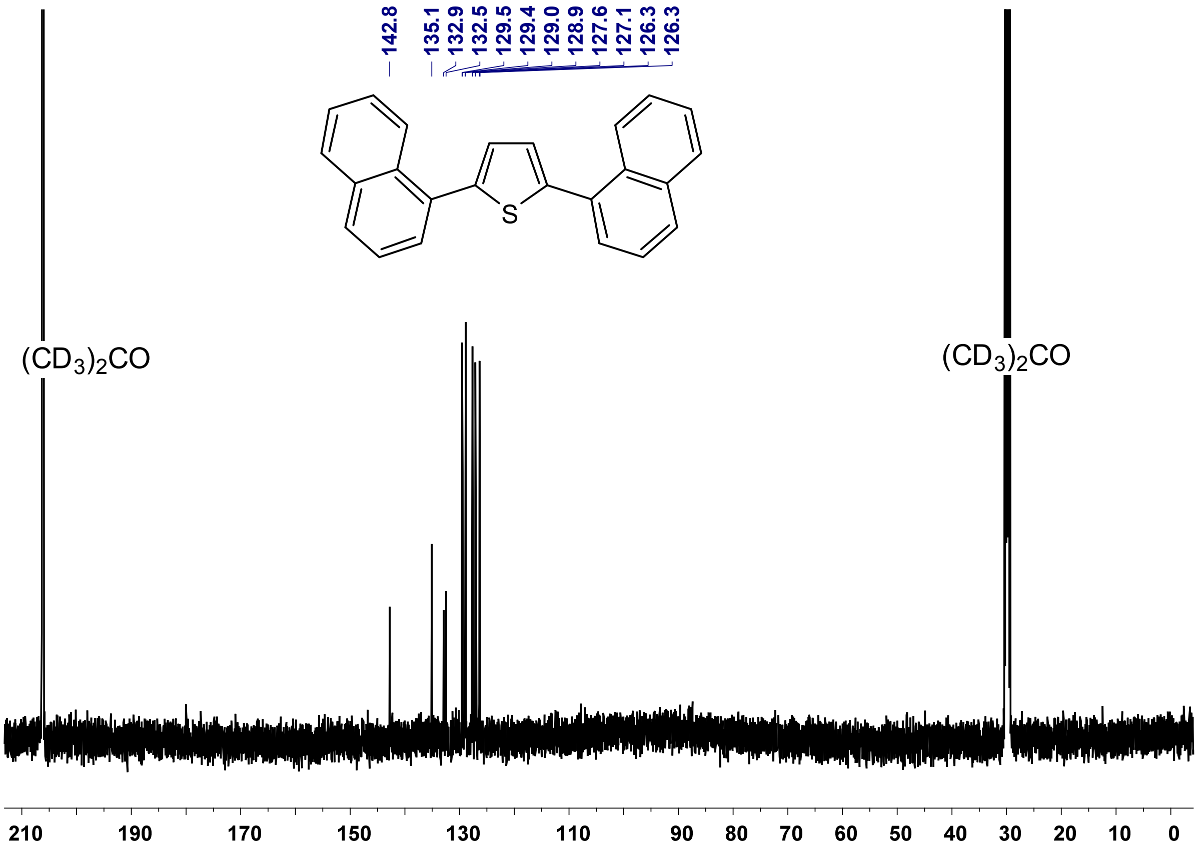

^{13}C NMR spectrum of **2l** recorded in acetone-d_6 at 298 K (δ in ppm).

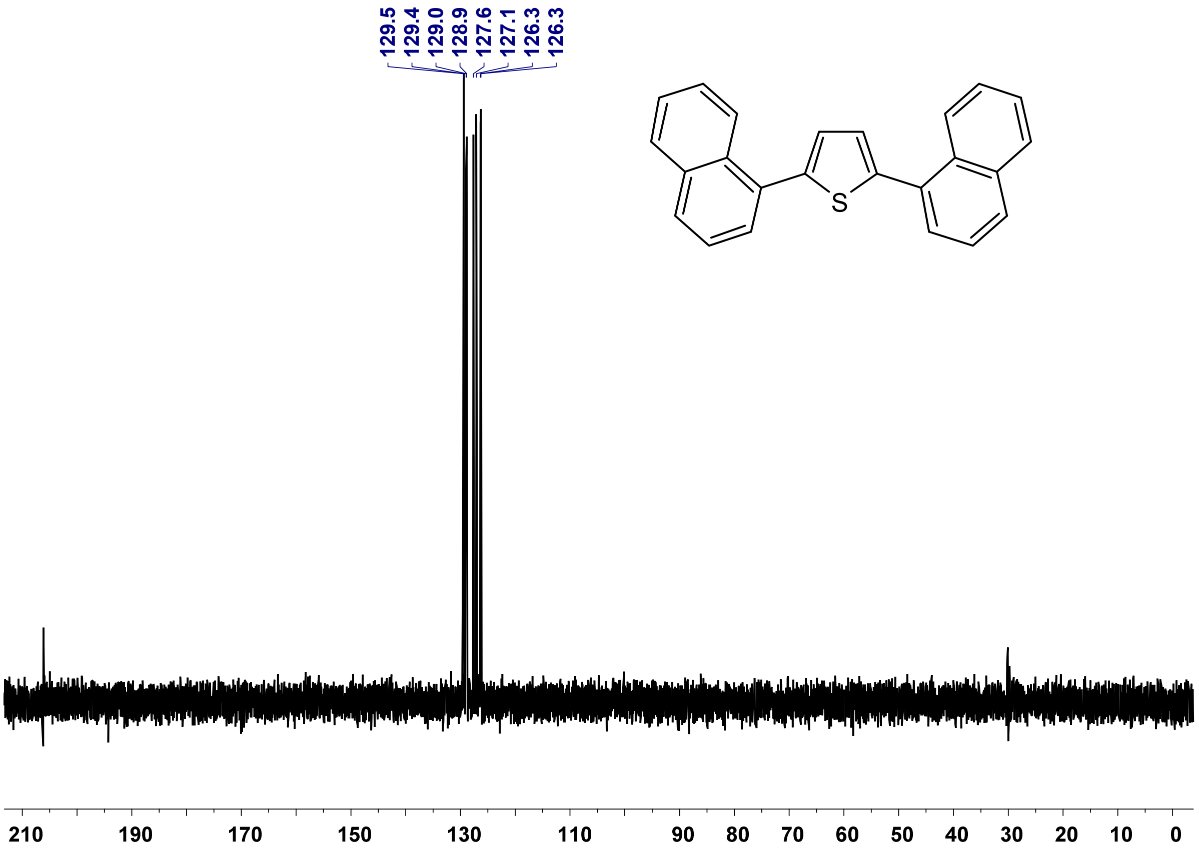

^{13}C DEPT-135 NMR spectrum of **2l** recorded in acetone-d_6 at 298 K (δ in ppm).

2,5-di(1*H*-indol-3-yl)thiophene (2p)

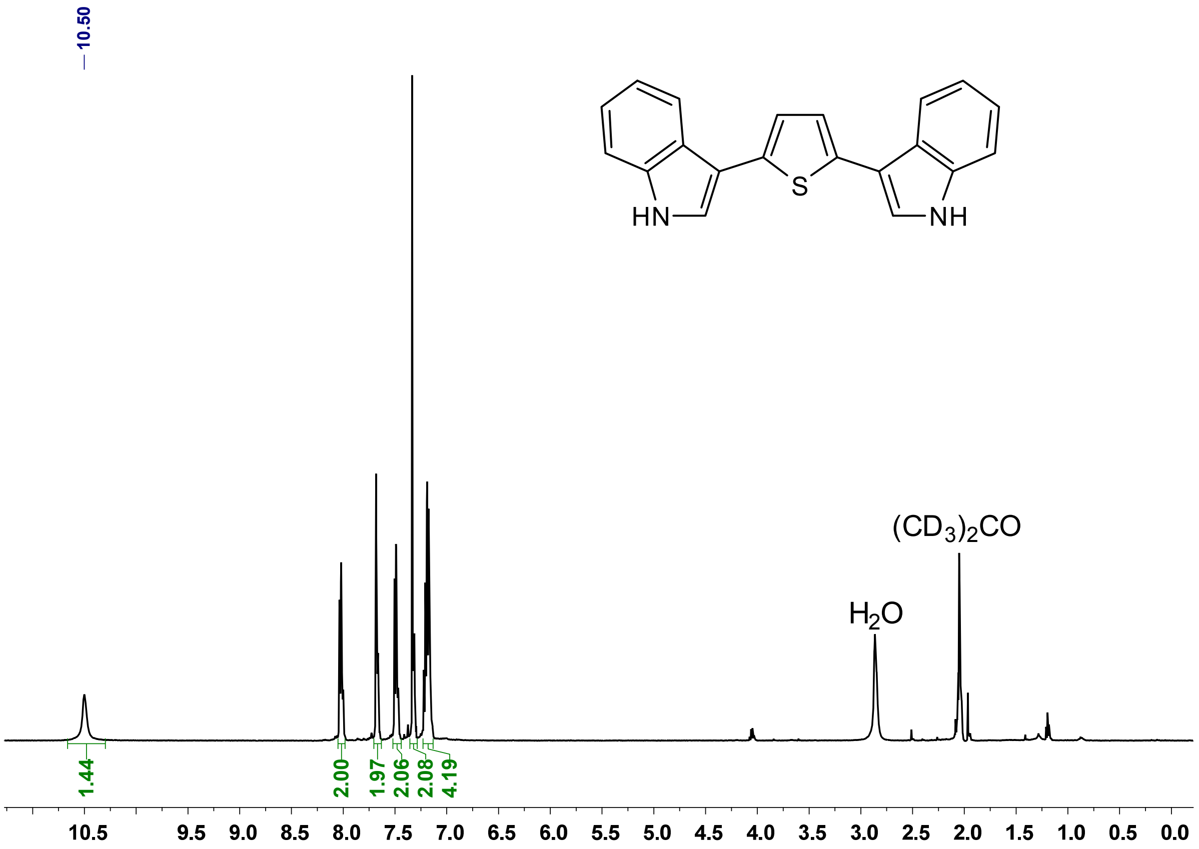

^{1}H NMR spectrum of **2p** recorded in acetone-d_6 at 298 K (δ in ppm).

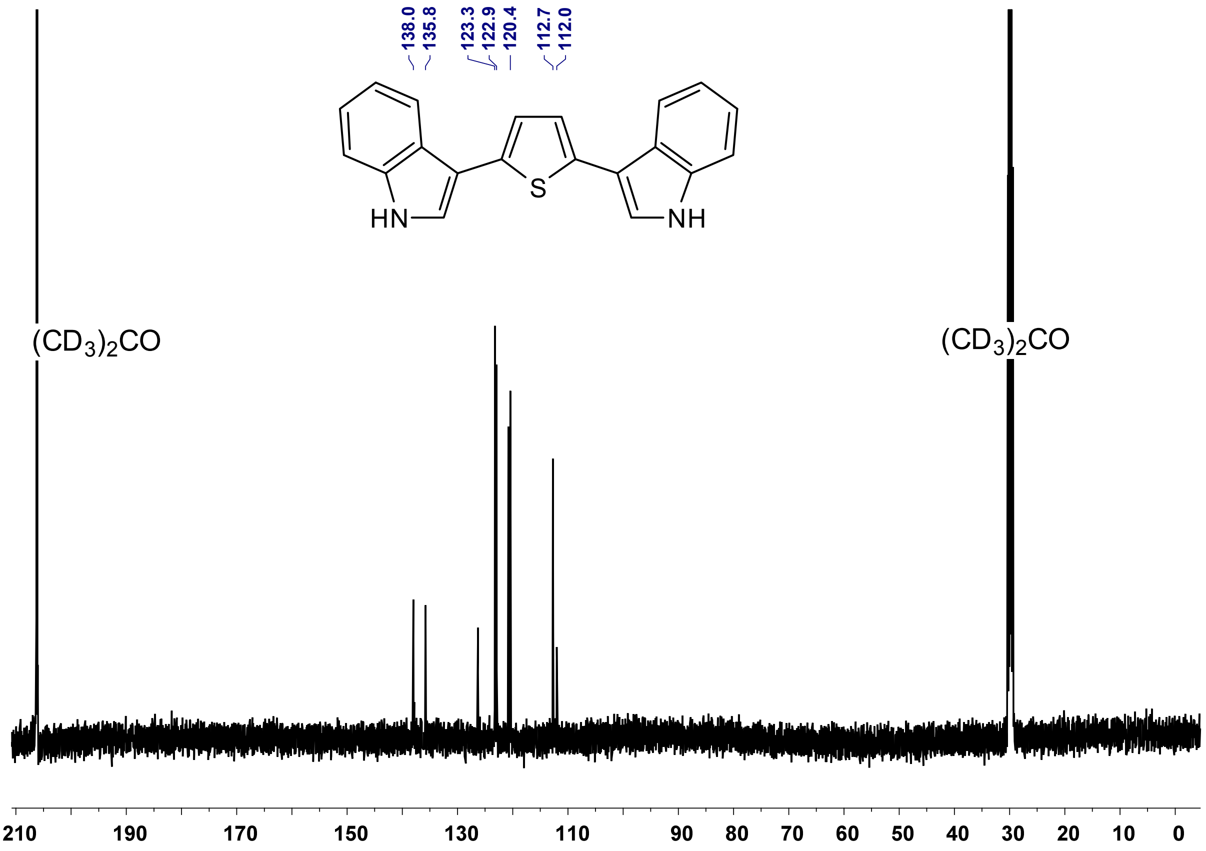

^{13}C NMR spectrum of **2p** recorded in acetone-d$_6$ at 298 K (δ in ppm).

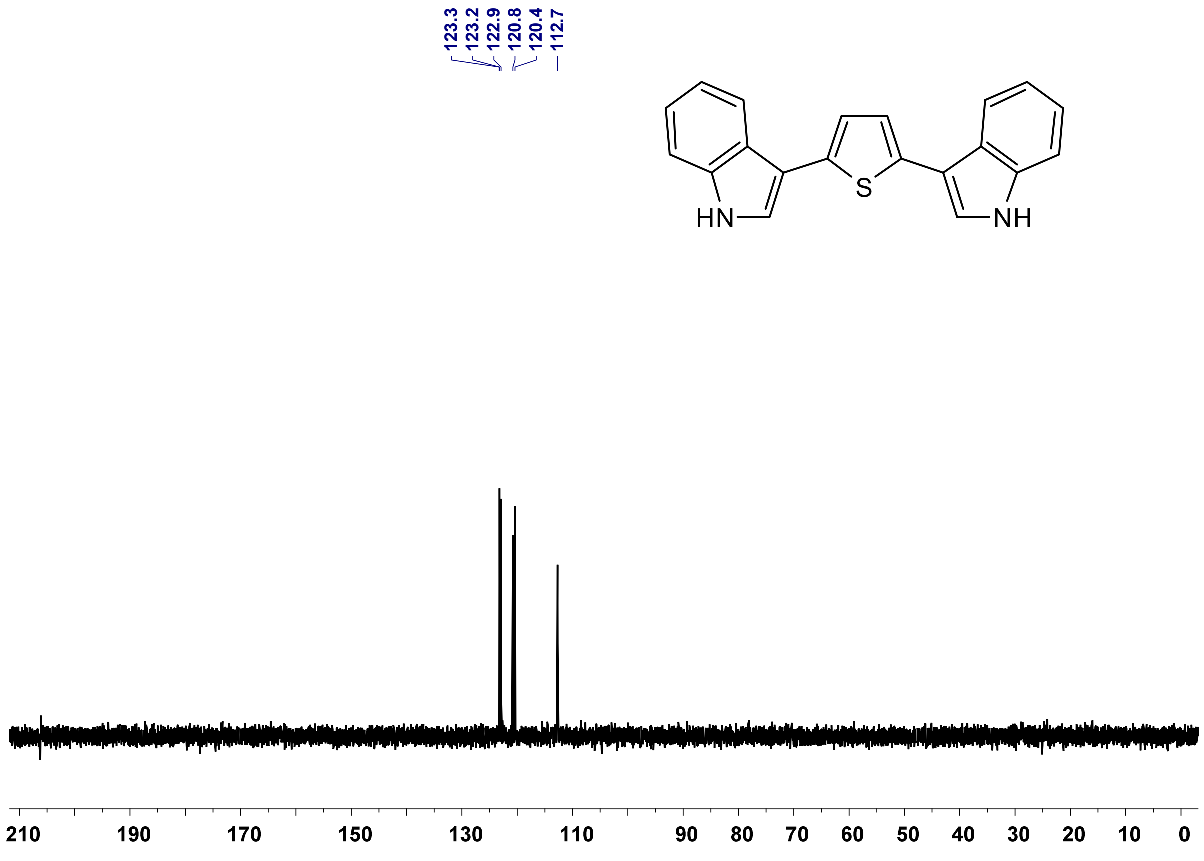

^{13}C DEPT-135 NMR spectrum of **2p** recorded in acetone-d$_6$ at 298 K (δ in ppm).
